# Estrogen receptor variant ERα46 and insulin receptor drive in primary breast cancer cells growth effects and interleukin 11 induction prompting the motility of cancer‐associated fibroblasts

**DOI:** 10.1002/ctm2.516

**Published:** 2021-11-04

**Authors:** Francesca Cirillo, Michele Pellegrino, Marianna Talia, Ida Daniela Perrotta, Damiano Cosimo Rigiracciolo, Asia Spinelli, Domenica Scordamaglia, Lucia Muglia, Rita Guzzi, Anna Maria Miglietta, Ernestina Marianna De Francesco, Antonino Belfiore, Marcello Maggiolini, Rosamaria Lappano

**Affiliations:** ^1^ Department of Physics University of Calabria Rende Italy; ^2^ Department of Pharmacy, Health and Nutritional Sciences University of Calabria Rende Italy; ^3^ Centre for Microscopy and Microanalysis, Transmission Electron Microscopy Laboratory, and Department of Biology, Ecology and Earth Sciences University of Calabria Rende Italy; ^4^ Breast Unit Regional Hospital Cosenza Cosenza Italy; ^5^ Department of Clinical and Experimental Medicine, University of Catania Garibaldi‐Nesima Hospital Catania Italy

**Keywords:** breast cancer, ERα46, estrogens, insulin, insulin receptor

## Abstract

Among the prognostic and predictive biomarkers of breast cancer (BC), the role of estrogen receptor (ER)α wild‐type has been acknowledged, although the action of certain ERα splice variants has not been elucidated. Insulin/insulin receptor (IR) axis has also been involved in the progression and metastasis of BC. For instance, hyperinsulinemia, which is often associated with obesity and type 2 diabetes, may be a risk factor for BC. Similarly, an aberrant expression of IR or its hyperactivation may correlate with aggressive BC phenotypes. In the present study, we have shown that a novel naturally immortalized BC cell line (named BCAHC‐1) is characterized by a unique expression of 46 kDa ERα splice variant (ERα46) along with IR. Moreover, we have shown that a multifaceted crosstalk between ERα46 and IR occurs in BCAHC‐1 cells upon estrogen and insulin exposure for growth and pulmonary metastasis. Through high‐throughput RNA sequencing analysis, we have also found that the cytokine interleukin‐11 (IL11) is the main factor linking BCAHC‐1 cells to breast cancer‐associated fibroblasts (CAFs). In particular, we have found that IL11 induced by estrogens and insulin in BCAHC‐1 cells regulates pro‐tumorigenic genes of the “extracellular matrix organization” signaling pathway, such as ICAM‐1 and ITGA5, and promotes both migratory and invasive features in breast CAFs. Overall, our results may open a new scientific avenue to identify additional prognostic and therapeutic targets in BC.

## Graphical abstract

– The crosstalk between ERα46 and IR signaling is involved in the growth and pulmonary metastasis of BCAHC‐1 cells.
– IL11 is the most induced gene by E2 and insulin stimulation in BCAHC‐1 cells, as assessed by high‐throughput RNA sequencing analysis.
– IL11 produced by BCAHC‐1 cells promotes gene expression changes and invasive features in CAFs

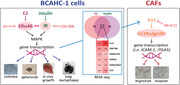

**Abbreviations**
BC: breast cancer
CAFs: cancer‐associated fibroblasts
CM: conditioned medium
E2: 17β‐estradiol
ECM: extracellular matrix
ERK1/2: extracellular signal‐regulated kinase 1/2
ERα46: 46 kDa ERα splice variant
ERα66: 66 kDa estrogen receptor α
FAP: fibroblast activation protein α
FBS: fetal bovine serum
ICAM‐1: intercellular adhesion molecule 1
IL11: interleukin‐11
IR: insulin receptor
IR‐A: insulin receptor isoform A
IR‐B: insulin receptor isoform B
ITGA5: integrin alpha 5
MAPK: mitogen‐activated protein kinase
OS: overall survival

## 1 BACKGROUND

Breast cancer (BC) is the most frequently diagnosed malignancy and the leading cause of tumor‐related death in women worldwide.^1^ Microarray‐based gene expression profiling has strongly contributed to the heterogenous characterization of BC, refining taxonomy and identifying novel prognostic and predictive gene signatures.^2^, ^3^ Moreover, a better understanding of the BC biological landscape has been achieved through several BC cell lines representing the molecular features of BC subgroups.^4^ Nonetheless, relevant cell models are still lacking for certain histopathological types and receptor expression patterns. In addition, concerns have been raised on the extent to which a long‐established cell line might replicate the initial biological settings.^4^

The multifaceted actions of estrogens are mainly mediated by estrogen receptor (ER)α and ERβ, which act as ligand‐activated transcription factors to regulate numerous estrogen‐responsive genes.^5^ The 66 kDa wild‐type full‐length ERα (ERα66) is expressed in approximately 70% of all BCs^6^, ^7^; however, ERα proteins encoded by mRNA splice variants have also been identified as further mediators of responses to estrogens.^8^ For instance, an exon‐1‐truncated ERα transcript with a molecular weight of 46 kDa (ERα46) has been found in various normal and tumor cell types, including BC.^9^, ^10^ ERα46 is characterized by the lack of the N‐terminus region containing the ligand‐independent AF‐1 transactivation domain.^9^ Biologically, ERα46 is involved in the growth arrest of cancer cells, interfering with the binding of ERα66 to DNA.^9^, ^11^ To date, the mechanisms triggering the unique responses mediated by ERα46, especially in BC, remain unclear.

In addition to estrogens, insulin signaling has been involved in BC progression, metastasis, and resistance to chemotherapeutics.^12^ For instance, the overexpression of the insulin receptor (IR) isoform A (IR‐A) contributes to metabolic reprogramming and development of BC.^13^ Notably, the bidirectional interaction between estrogen and insulin signals may generate a cross‐communication that results in aggressive and life‐threatening BC phenotypes.^14^, ^15^, ^16^ In this regard, estrogen‐activated ERα may prompt the insulin transduction pathway, and insulin stimulation may induce the ligand‐independent phosphorylation of ERα in BC cells.^15^, ^16^

The acquisition of aggressive and metastatic features of BC cells is facilitated by their multi‐layered interaction with the surrounding microenvironment.^17^, ^18^ Cancer‐associated fibroblasts (CAFs), which constitute a predominant cell component of the BC stroma, may serve as a source of hormones, growth factors, cytokines, and other mediators that promote the growth and motility of BC cells.^19^ Similarly, BC‐derived effectors sustain the growth of tumor cells and induce epigenetic and transcriptional changes that enable CAFs to acquire an active secretome for their aggressive phenotype.^19^, ^20^

Here, we provide unique evidence about a naturally immortalized BC cell line, named BCAHC‐1, which was derived from a patient with invasive ductal breast carcinoma. Notably, these cells are characterized by the unique expression and bi‐directional cooperation between ERα46 and IR for the growth and pulmonary metastasis upon estrogen and insulin stimulations. In addition, we show that the crosstalk between the aforementioned receptors triggers the expression and function of the cytokine interleukin‐11 (IL11), which leads to migratory and invasive capabilities of CAFs derived from BC patients. The latter findings were corroborated by evidence from BC datasets that ascertained the association of IL11 with poor outcomes and pro‐invasive pathways in BC patients.

## 2 METHODS

A detailed description of the reagents, in vivo and in vitro analyses as well as protocols for sample preparation, gene expression studies, RNA‐seq analysis, reporter gene assays and gene silencing experiments, western blot and co‐immunoprecipitation assays, immunofluorescence and electron microscopic studies, ELISA, proliferation, clonogenic and spheroid formation assays, bioinformatics, and statistical procedures can be found in Supplementary Materials and Methods.

### 2.1 Cell cultures

#### 2.1.1 BCAHC‐1 cells

BCAHC‐1 cells were isolated from a 45‐year‐old woman with high grade infiltrating ductal carcinoma (ER‐positive, PR and HER2‐negative, Ki‐67‐positive). The patient had no pregnancy, did not use hormone therapy, and was in good general health until diagnosis. A small tissue sample was minced into approximately 1–2 mm^3^ pieces, washed with phosphate‐buffered saline (PBS), and placed in Accumax cell detachment solution (Merck Life Science, Milan, Italy) for 45 min at room temperature with gentle but constant mixing. After letting the remaining tissue pieces settle down (for 2 min), the supernatant was progressively filtered using cell strainers of descending pore size down to 40 μm to obtain a single‐cell suspension. This suspension was then diluted with Dulbecco's Modified Eagle Medium: Nutrient Mixture F‐12 (DMEM/F‐12, Life Technologies, Milan, Italy) supplemented with 5% fetal bovine serum (FBS) and centrifuged at 240×g for 10 min. The pellet obtained was suspended in DMEM/F‐12 supplemented with 5% FBS and 100 μg/ml penicillin/streptomycin and incubated at 37°C in a humidified 5% CO_2_ atmosphere. After 6 days, a fresh medium was added, and after 10–14 days, small aggregates of fewer than 10 cells appeared. An outgrowth of cells attached to the plate was observed; later, epithelial‐like cells started to make colonies in dome‐like shapes. These cell domes were isolated, picked, and transferred to 10‐cm Petri dishes. The cells were regularly passaged and expanded in the above‐indicated medium at 37°C in a humidified 5% CO_2_ atmosphere. Cells were characterized by immunofluorescence and flow cytometry (Supplementary Materials and Methods) with anti‐cytokeratin‐FITC (IM2356U, Beckman Coulter, Milan, Italy) and anti‐FAPα antibodies (H‐56, Santa Cruz Biotechnology, DBA, Milan, Italy). BCAHC‐1 cells were deposited at DSMZ (Leibniz Institute DSMZ‐German Collection of Microorganisms and Cell Cultures) and patented (n. 102019000022167).

#### 2.1.2 CAFs

CAFs were obtained as previously described^21^ from 10 invasive ductal breast carcinomas and pooled for the subsequent studies. Briefly, specimens were cut into 1–2 mm diameter pieces, placed in a digestion solution comprising 400 IU collagenase, 100 IU hyaluronidase, 10% serum, antibiotics, and antimycotics, and incubated overnight at 37 °C. After centrifugation at 90×g for 2 min, the supernatant containing fibroblasts was centrifuged at 485×g for 8 min; the pellet obtained was suspended in Medium 199 and Ham's F12 mixed 1:1 (supplemented with 10% FBS and 100 μg/ml penicillin/streptomycin). CAFs were then expanded into 10‐cm Petri dishes and stored as cells passaged for three population doublings within a total of 7 to tissue dissociation. Primary cell cultures of fibroblasts were characterized by immunofluorescence with human anti‐vimentin (V9) and human anti‐cytokeratin 14 (LL001) (Santa Cruz Biotechnology, DBA, Milan, Italy) (data not shown). FAPα antibody (H‐56, Santa Cruz Biotechnology, DBA, Milan, Italy) was used to characterize activated fibroblasts (data not shown). We used CAFs passaged for up to 10 population doublings for the experiments to minimize clonal selection and culture stress, which could occur during extended tissue culture.

#### 2.1.3 Cell lines

MCF‐7, SkBr3 and T47D breast and LNCaP prostate cancer cells were obtained from ATCC (Manassas, VA, USA), used less than 6 months after resuscitation, routinely tested, and authenticated according to the ATCC suggestions. MCF‐7 BC cells were maintained in DMEM/F‐12 with phenol red, supplemented with 10% FBS and 100 μg/ml penicillin/streptomycin. LNCaP prostate and SkBr3 BC cells were maintained in RPMI 1640, with and without phenol red, respectively, supplemented with 10% FBS and 100 mg/ml penicillin/streptomycin. T47D cells were maintained in RPMI 1640 with phenol red supplemented with 10% FBS, 0.2 Units/ml bovine insulin (Sigma‐Aldrich) and 100 mg/ml penicillin/streptomycin.

Cells were grown in a 37°C incubator with 5% CO_2_ and switched to a medium without serum and phenol red the day before treatments to be processed for experiments.

### 2.2 In vivo studies

Female 45‐day‐old athymic nude mice (nu/nu Swiss, Envigo, Udine, Italy) were maintained in a sterile environment. On day 0, exponentially growing BCAHC‐1 cells (2 × 10^6^ per mouse) were implanted in mammary fat pad in 0.1 ml of Matrigel (Cultrex, Trevigen, Mediemme srl, Cosenza, Italy). When the tumors reached an average ∼0.15 cm^3^ (in about 1 week), mice were randomly allocated to nine groups (*n* = 6) according to treatments administered for 28 days by subcutaneous injection (vehicle, E2, insulin, and ICI) or by gavage (OSI‐906), as detailed in Supplementary Materials and Methods. BCAHC‐1 xenograft tumor growth was measured twice a week by caliper, along two orthogonal axes: length (L) and width (W). Tumor volumes (in cubic centimeters) were estimated by the following formula: TV = L × [W2]/2. At 28 days of treatment, the animals were sacrificed following the standard protocols, and tumors were dissected from the neighboring connective tissue. Half of each tumor was fixed in 10% formalin for 24 h prior to paraffin‐embedding for the subsequent histologic analyses. Animal care, death, and experiments were performed in accordance with the principle of the 3Rs and according to Italian law (D.L. 26/2014), the Guide for Care and Use of Laboratory Animals published by the US National Institutes of Health (2011), and the Directive 2010/63/EU of the European Parliament on the protection of animals used for Scientific research. Histologic analysis and immunohistochemistry were performed as described in Supplementary Materials and Methods.

### 2.3 Lung metastasis experiment

Female 45‐day‐old athymic nude mice were maintained as described above. On day 0, exponentially growing BCAHC‐1 cells (1 × 10^6^ per mouse) were suspended in 100 μl of PBS and injected into the tail veins. The next day, mice were randomly allocated to nine groups and treated as described in Supplementary Materials and Methods. At the end of treatment (days 15), all mice were sacrificed and analyzed by India ink assay for metastatic disease. Pulmonary metastases were enumerated by intra‐tracheal injection of India ink (15% India ink, 85% water, and 3 drops NH4OH/100 ml). India ink injected lungs were washed in Feket's solution (300 ml 70% EtOH, 30 ml 37% formaldehyde, and 5 ml glacial acetic acid) and then placed in fresh Feket's solution overnight. The white metastatic nodules against a dark lung background were counted and photographed.

## 3 RESULTS

### 3.1 Characterization of BCAHC‐1 BC cells

**FIGURE 1:**
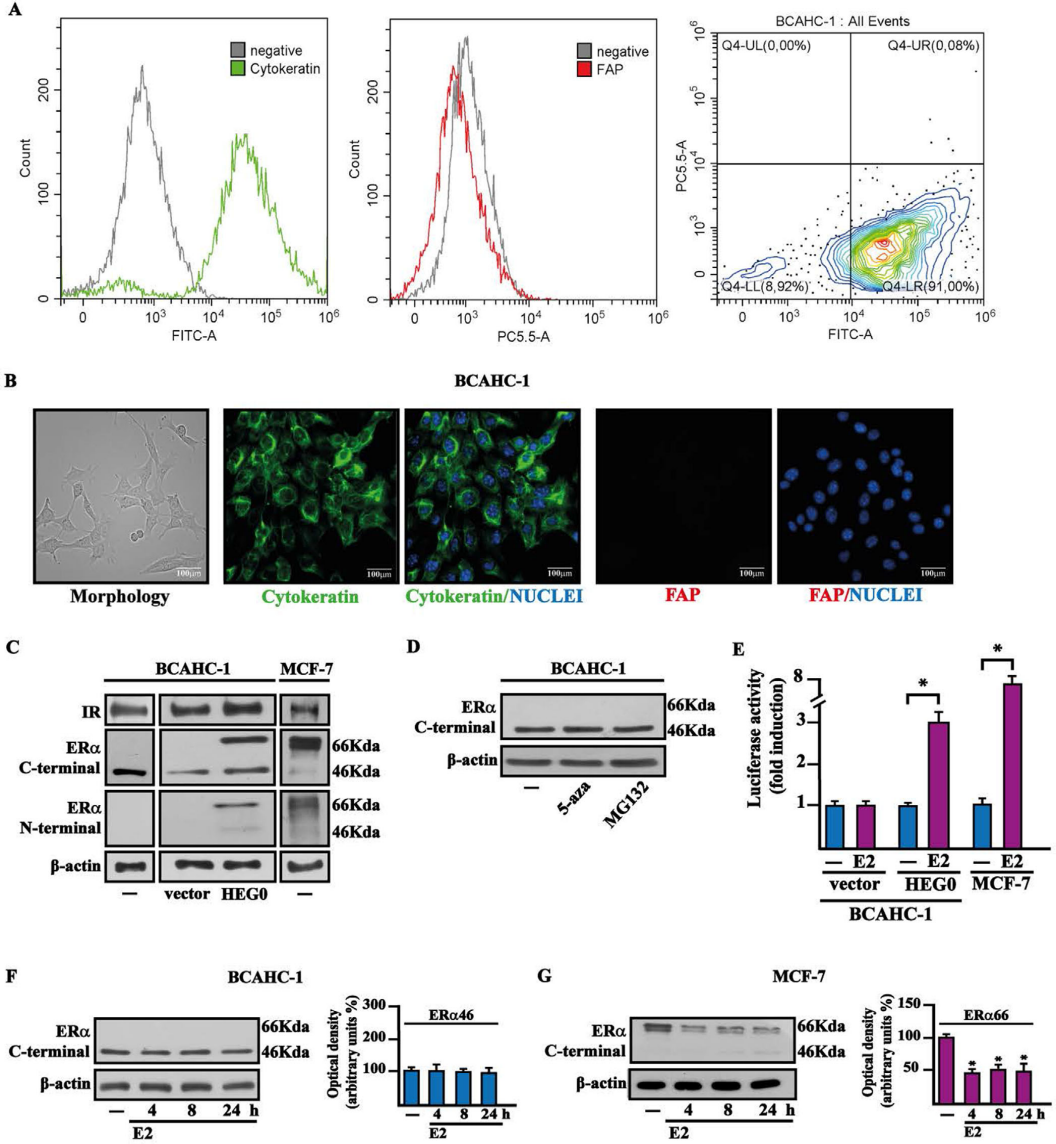
Molecular features of BCAHC‐1 cells. (A) Expression of cytokeratin and fibroblast activation protein (FAP) in BCAHC‐1 cells, as shown by flow cytometry. Representative overlay histograms and dot plot showing cytokeratin‐positive (green) and FAP‐negative (red) BCAHC‐1 cells. FITC, fluorescein isothiocyanate; PE‐Cy5.5, phycoerythrin‐Cyanin 5.5. (B) Morphological appearance of BCAHC‐1 cells in phase‐contrast microscopy, cytokeratin‐positive (green signal), and FAP‐negative (red signal) immunofluorescence staining in BCAHC‐1 cells. Nuclei were stained by DAPI (blue signal). Scale bar: 100 μm. (C) Immunoblots of IR, ERα66, and ERα46 in BCAHC‐1 and MCF‐7 breast cancer cells, as indicated. BCAHC‐1 cells were transiently transfected with an empty vector (vector) or the wild‐type ERα (HEG0). (D) Protein levels of ERα46 in BCAHC‐1 cells exposed for 24 h to 10 μM DNA‐hypomethylating agent 5‐aza‐2′‐deoxycytidine (5‐aza) and proteasome inhibitor MG132. β‐Actin was used as a loading control. (E) BCAHC‐1 and MCF‐7 cells were transfected with the ER luciferase reporter plasmid ERE‐luc combined with an empty vector (vector) or the wild‐type ERα (HEG0) for 8 h and treated with 100 nM E2 for 18 h, as indicated. The luciferase activities were normalized to the internal transfection control, and values of cells receiving vehicle (−) were set as 1‐fold induction on which the activity induced by E2 was calculated. Columns represent the mean ± SD of three independent experiments performed in triplicate. Protein levels of ERα46 in BCAHC‐1 (F) and ERα66 in MCF‐7 (G) cells treated with vehicle (−) or 100 nM E2 for the indicated times. Side panels show densitometric analysis of the blots normalized to β‐actin. Values represent the mean ± SD of three independent experiments performed in triplicate. (*) indicates *p* < 0.05 for cells treated with E2 relative to cells treated with vehicle (−) Abbreviations: E2, 17β‐estradiol; ERα46, 46 kDa ERα splice variant; ERα66, 66 kDa estrogen receptor α; FAP, fibroblast activation protein; IR, insulin receptor; SD, standard deviation.

**FIGURE 2:**
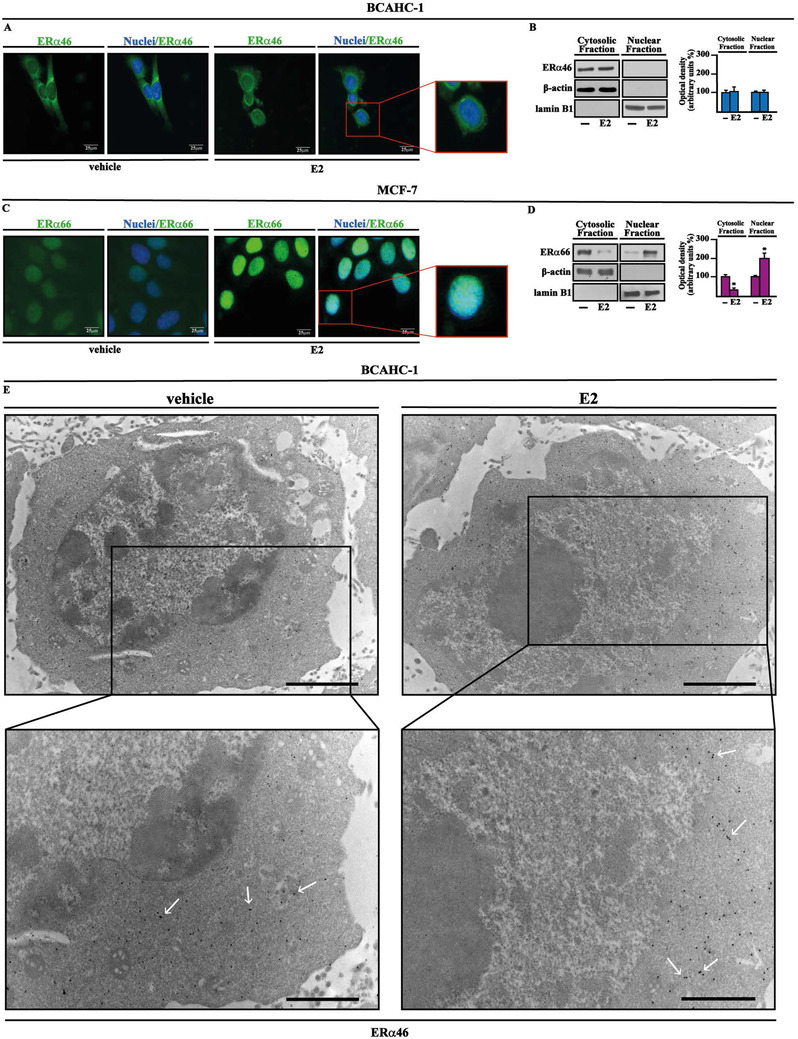
Cytoplasmic localization of ERα46 in BCAHC‐1 cells. Representative images of BCAHC‐1 (A) and MCF‐7 (C) cells immunostained with an ERα C‐terminal antibody (green signal). Cells were treated with vehicle or 100 nM E2 for 1 h, and nuclei were stained by DAPI (blue signal). The images shown represent 10 random fields from three independent experiments. Scale bar: 25 μm. Enlarged details are shown in the separate boxes. Immunoblots (using an ERα C‐terminal antibody) of cytosolic and nuclear fraction lysates derived from BCAHC‐1 (B) and MCF‐7 (D) cells treated for 1 h with vehicle (−) or 100 nM E2, as indicated. Side panels show densitometric analysis of the blots normalized to lamin B1 or β‐actin, which served as a nuclear or cytoplasmic marker, respectively. Values represent the mean ± SD of three independent experiments performed in triplicate. (*) indicates *p* < 0.05 for cells treated with E2 relative to cells treated with vehicle (−). (E) Immunoelectron microscopic localization of ERα46 (as indicated by representative arrows) in BCAHC‐1 cells treated for 1 h with vehicle or 100 nM E2. Enlarged details are shown in the lower boxes. Scale bar of the upper boxes: 2.0μm. Scale bar of the lower boxes: 1.0μm Abbreviations: ERα46, 46 kDa ERα splice variant; E2, 17β‐estradiol; SD, standard deviation.

We began the present study by successfully establishing BCAHC‐1 cells in vitro from a surgically resected high‐grade infiltrating ductal carcinoma. Flow cytometry analyses (Figure 1A) and immunofluorescent staining (Figure 1B) revealed that BCAHC‐1 cells express the epithelial marker cytokeratin but not the fibroblast activation protein α (FAP) that represents a CAFs surface marker. Microscopic evaluation showed that BCAHC‐1 cells are spindle in shape, grow mainly in tight clusters as adherent colonies, and exhibit an epithelial‐like morphology (Figure 1B). Consistent with immortalized cancer cells, BCAHC‐1 cells exhibited high levels of telomerase activity relative to primary non‐immortalized CAFs, as determined by TRAPeze assay (data not shown). Then, real‐time PCR experiments were performed to investigate the expression profile of hormone and growth factor receptors. Notably, we showed that BCAHC‐1 cells are characterized by a unique expression of ERα using primers directed against the C‐E domains, along with the predominant expression of IR isoform A relative to the IR isoform B (Table S1). Immunoblots of BCAHC‐1 cells confirmed IR positivity and ERα46 expression as assessed using an ERα C‐terminal antibody, whereas MCF‐7 BC cells expressed both ERα 46 kDa and 66 kDa isoforms (Figure 1C). Corroborating the aforementioned findings, western blots performed using an ERα N‐terminal antibody showed a 66 kDa band only in BCAHC‐1 cells transfected with a construct encoding the wild‐type ERα (HEG0) (Figure 1C). Next, the DNA‐hypomethylating agent 5‐aza‐2′‐deoxycytidine (5‐aza) and the proteasome inhibitor MG132 did not affect the expression of ERα66 in BCAHC‐1 cells (Figure 1D), suggesting that DNA methylation and proteasomal degradation events are not implicated in the lack of ERα66 expression in BCAHC‐1 cells. To evaluate whether ERα46 can mediate the transcriptional response upon 17β‐estradiol (E2) treatment, BCAHC‐1 cells were transfected with the ER reporter plasmid (ERE‐luc). We did not observe luciferase induction that was detected in MCF‐7 cells or co‐transfecting HEG0 in BCAHC‐1 cells (Figure 1E). In addition, E2 did not induce regulatory effects on the mRNA levels of ER target genes namely TFF1, cathepsin D and progesterone receptor (PR), contrary to what observed in MCF‐7 cells (Figure S1A). As the down‐regulation of ERα66 by estrogens has been considered a hallmark for receptor activation,^22^ we also investigated whether E2 modulates the protein levels of ERα46. Remarkably, E2 did not modify ERα46 levels in BCAHC‐1 cells (Figure 1F), whereas E2 downregulated the ERα66 levels in MCF‐7 cells (Figure 1G). Considering the unique expression of ERα46 and IR exhibited by BCAHC‐1 cells, we aimed to provide additional insights into their responses to the cognate ligands E2 and insulin, respectively. In this regard, we first noted the accumulation of ERα46 in the cytoplasmic compartment of BCAHC‐1 cells upon exposure to E2, as shown by immunofluorescence and subcellular fractionation studies (Figure 2A,B). In contrast, E2 largely triggered the localization of ERα66 within the nuclear compartment of MCF‐7 cells (Figure 2C,D), as previously reported.^23^ Electron microscopy experiments confirmed that ERα46 is located in the cytoplasm of BCAHC‐1 cells upon E2 stimulation (Figure 2E).

### 3.2 Functional crosstalk between ERα46 and IR in BCAHC‐1 cells

**FIGURE 3:**
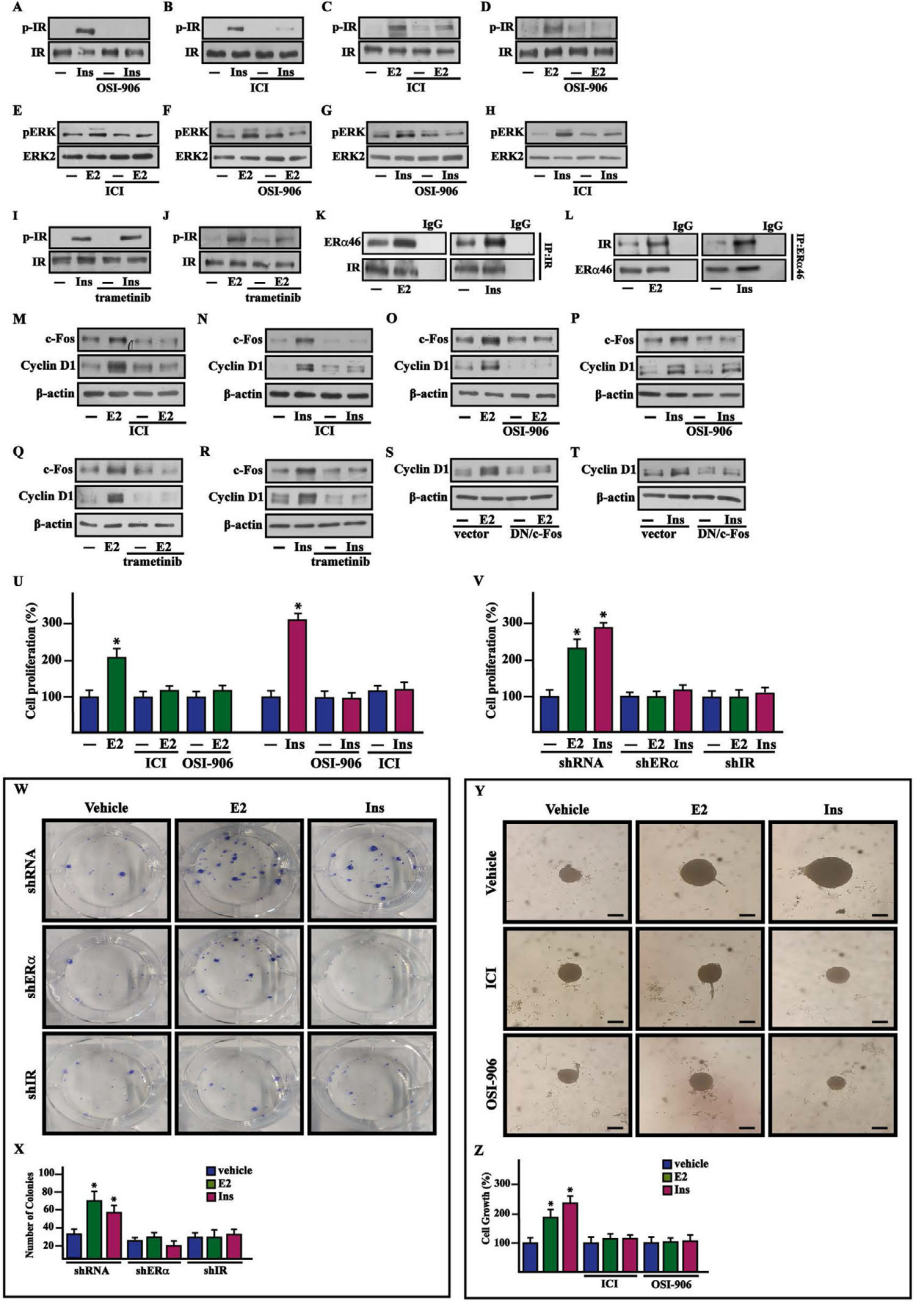
The crosstalk between ERα46 and IR signaling is involved in the growth of BCAHC‐1 cells induced by E2 and insulin. Protein levels of pIR and pERK in BCAHC‐1 cells exposed for 10 min to 10 nM insulin (Ins) (A and B and G and H) and 100 nM E2 (C and D and E and F) in the presence or absence of 1 μM IR inhibitor (OSI‐906) and ERα antagonist ICI 182,780 (ICI), as indicated. Protein levels of pIR in BCAHC‐1 cells treated for 10 min with 10 nM insulin (Ins) (I) and 100 nM E2 (J) alone or in combination with 100 nM MEK inhibitor trametinib. (K and L) Co‐immunoprecipitation assays performed in BCAHC‐1 cells treated for 1 h with 100 nM E2 and 10 nM insulin (Ins). Cell lysates were immunoprecipitated with anti‐IR (K) or anti‐ERα (L) antibodies. Immunocomplexes were analyzed by immunoblot with antibodies against the indicated proteins. In control samples, nonspecific IgG was used instead of the primary antibody. Immunoblots of c‐Fos and Cyclin D1 from BCAHC‐1 cells treated for 8 h with vehicle (−), 100 nM E2 or 10 nM insulin (Ins) alone or in combination with 1 μM ERα antagonist ICI 182,780 (ICI) (M‐N), 1μM IR inhibitor OSI‐906 (O‐P) or 100 nM MEK inhibitor trametinib (Q and R). Immunoblots of Cyclin D1 from BCAHC‐1 cells transfected for 24 h with a vector or a dominant‐negative c‐Fos construct (DN/c‐Fos) and then exposed for 8 h to 100 nM E2 (S) or 10 nM insulin (Ins) (T). The proliferative effects observed in BCAHC‐1 cells following 5 days treatment with 100 nM E2 and 10 nM insulin (Ins) are prevented by 1μM ERα antagonist ICI 182,780 (ICI) and 1 μM IR inhibitor OSI‐906 (U) as well as knocking down ERα46 and IR expression (V). The proliferation of cells treated with vehicle (−) was set as 100% upon which cell growth induced by treatments was calculated. Each data point is the mean ± SD of three independent experiments performed in triplicate. (W) Colony formation assay in BCAHC‐1 cells exposed to vehicle, 100 nM E2 or 10 nM insulin (Ins) transfected every 2 days as indicated. After 10 days of incubation, the plates were stained with Giemsa, and colonies were counted as reported in panel X. (Y) Representative images of spheroids (a single spheroid per well) grown on agar‐coated plates upon 20 days treatment with vehicle, 100 nM E2 or 10 nM insulin (Ins) alone or in combination with 1 μM ERα antagonist ICI 182,780 (ICI) and 1 μM IR inhibitor OSI‐906, as indicated. Scale bar: 100 μm. (Z) Quantification of cell growth. Vehicle (−) was set as 100% upon which the growth induced by treatments was calculated. Each column represents the mean ± SD of three independent experiments, each performed in triplicate. (*) indicates *p*  <  0.05 for cells receiving treatments versus vehicle (−) Abbreviations: ERα46, 46 kDa ERα splice variant; E2, 17β‐estradiol; IR, insulin receptor; SD, standard deviation.

Based on the aforementioned results and considering that estrogens cooperate with insulin signaling for BC progression and metastasis,^14^, ^15^, ^16^ we aimed to evaluate whether such crosstalk may also occur in BCAHC‐1 cells. We found that IR phosphorylation induced by insulin is abrogated using the IR inhibitor OSI‐906 (Figures 3A and S1B) and the ERα antagonist ICI 182,780 (ICI) (Figures 3B and S1B). Besides, we obtained similar results after treating BCAHC‐1 cells with E2 (Figures 3C,D and S1B), in line with previous studies showing that estrogens may activate IR.^16^, ^24^ As the MAPK pathway triggers the transduction responses mediated by both estrogen and insulin signaling in cancer cells,^14^, ^16^ we noted that either ICI (Figure 3E,H and S1C) or OSI‐906 (Figures 3F,G and S1BC) prevents the ERK1/2 phosphorylation by both E2 and insulin in BCAHC‐1 cells. Moreover, we showed that the MEK inhibitor trametinib prevents IR activation prompted by E2 but not insulin (Figures 3I,J and S1D); hence, MEK signaling is involved in IR phosphorylation induced by E2 treatment. Through co‐immunoprecipitation assays, we ascertained that a physical interaction between ERα46 and IR is stimulated by both E2 and insulin in BCAHC‐1 cells (Figures 3K,L and S1E,F). Cumulatively, our findings suggest that these ligands trigger crosstalk between ERα46 and IR in BCAHC‐1 cells. Consistent with previous studies showing that ERK1/2 transduction pathway mediates the expression of estrogen and insulin target genes, such as c‐Fos and Cyclin D1,^25^, ^26^ we established that both E2 and insulin induce their mRNA (Figure S1G,H) and protein levels (Figures 3 M,R and S1I‐SK) in BCAHC‐1 cells. Notably, the protein increase of c‐Fos and Cyclin D1 triggered by both E2 and insulin was inhibited using ICI (Figures 3M,N, and S1I), OSI‐906 (Figures 3O,P and S1J), and trametinib (Figures 3Q,R and S1K). Considering that c‐Fos has been involved in Cyclin D1 regulation,^27^ we showed that Cyclin D1 induction by either E2 or insulin is prevented by transfecting BCAHC‐1 cells with the DN/c‐Fos expression vector (Figures 3S,T and S1L).

### 3.3 ERα46 and IR are involved in the growth and metastasis of BCAHC‐1 cells

**FIGURE 4:**
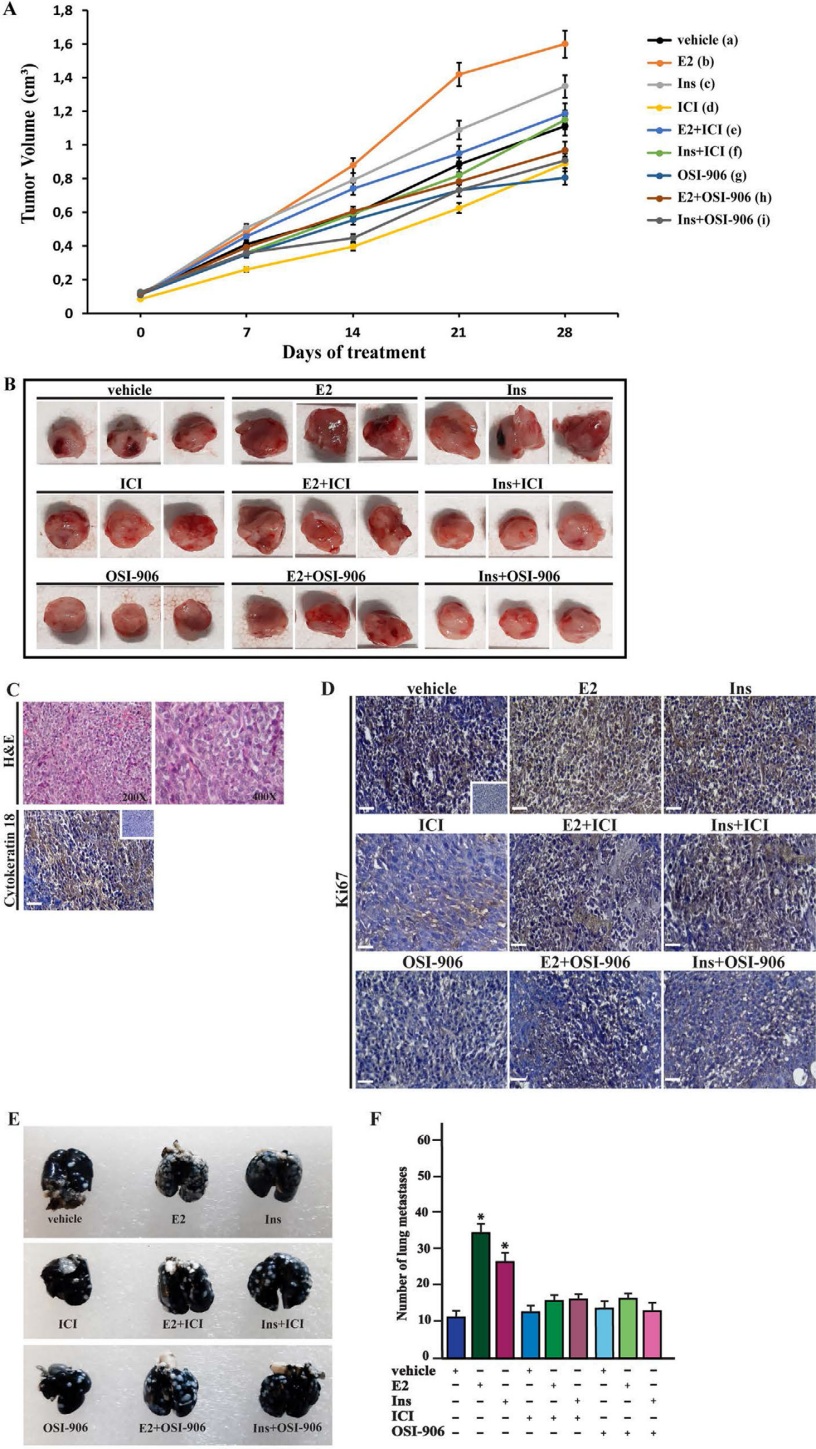
E2 and insulin induce the growth of BCAHC‐1‐derived xenografts and lung metastases. (A) Tumor volume from BCAHC‐1 xenografts implanted in female athymic nude mice treated for 28 days with vehicle, E2, insulin (Ins), ICI 182,780 (ICI), OSI‐906 alone or in combination (see details in Supplementary Materials and Methods). Tumor growth was monitored by caliper measuring the visible tumor sizes at indicated time points. Vehicle (a); E2 (b); Ins (c); ICI (d); OSI‐906 (e); E2+ICI (f); E2+OSI‐906 (g); Ins+ICI (h); Ins+OSI‐906 (g). *p* < 0.05 at the end of experiment among the following groups: b versus a; c versus a; d versus a; e versus a; f versus b; g versus b; h versus c; i versus c. (B) At the end of experiment, tumors were explanted, and for each group of animals (*n* = 6), three representative images of tumors are shown. (C) Formalin‐fixed paraffin‐embedded (FFPE) sections of tumor xenografts were stained with hematoxylin and eosin Y (H&E), and the epithelial nature of the tumors was verified by immunostaining with anti‐human cytokeratin 18 antibody. Scale bar: 25 μm. Insert: negative control. (D) The expression of Ki‐67, as a marker of proliferation, was evaluated in FFPE sections of explanted tumors from BCAHC‐1 xenografts treated as described in Supplementary Materials and Methods. Scale bar: 25 μm. Insert: negative control. (E) Representative photographs of lung metastases (white dots) after 15 days of treatment with vehicle, E2, insulin (Ins), ICI 182,780 (ICI), OSI‐906 alone or in combination (see Supplementary Materials and Methods). Lungs of xenografts were injected with India ink solution (15%) and fixed in Fekete's solution. (F) Enumeration of pulmonary metastatic nodules. The results are presented as mean ± SD (*n* = 6 lungs). (*) indicates *p* < 0.05 for animals treated with E2 and insulin (Ins) versus animals treated with vehicle Abbreviations: E2, 17β‐estradiol; FFPE, formalin‐fixed paraffin‐embedded; SD, standard deviation.

In line with the aforementioned data, we found that the proliferation and colony‑forming ability of BCAHC‐1 cells are stimulated by E2 and insulin through both ERα46 and IR, as demonstrated using ICI and OSI‐906 as well as silencing the expression of these receptors (Figures 3U‐3X and S1M and S2A,B). Moreover, the spheroid expansion observed upon E2 and insulin treatment in BCAHC‐1 cells was prevented using ICI and OSI‐906 (Figure 3Y,Z). Based on an in vivo model system, 45‐day‐old female nude mice were injected with BCAHC‐1 cells into the mammary fat pad region and treated with E2 and insulin alone and in combination with ICI and OSI‐906. The treatment with these compounds was well tolerated as we did not notice changes in body weight, food and water consumption, and motor function. In addition, the mean weight or histologic features of the major organs (liver, lung, spleen, and kidney) did not show differences among vehicle and compounds‐treated mice after sacrifice, thus indicating a lack of toxic effects. Notably, treatment with either ICI or OSI‐906 prevented tumor growth induced by E2 and insulin (Figure 4A,B). The epithelial nature of the tumors was verified by immunostaining, which revealed strong positivity for the human cytokeratin 18 (Figure 4C). Upregulated expression of the proliferative marker Ki67 was found in tumor tissue sections obtained from E2 and insulin‐treated mice relative to those exposed to ICI or OSI‐906 either alone or in combination with E2 and insulin (Figure 4D). Besides, BCAHC‐1 cells were injected into the tail veins of female athymic nude mice, and 15 days after injection, lungs were perfused with India ink to visualize lung metastases, which appear as macroscopic white colonies on a dark background (Figure 4E). Remarkably, E2 and insulin significantly increased the pulmonary metastatic nodules, which were lowered using ICI and OSI‐906 (Figure 4E,F). Taken together, these findings indicate that E2 and insulin stimulate BCAHC‐1 cell growth and metastatic spread through the action of both ERα46 and IR.

### 3.4 Transcriptome profiling of BCAHC‐1 cells identifies IL11 as a main target of both E2 and insulin

**FIGURE 5:**
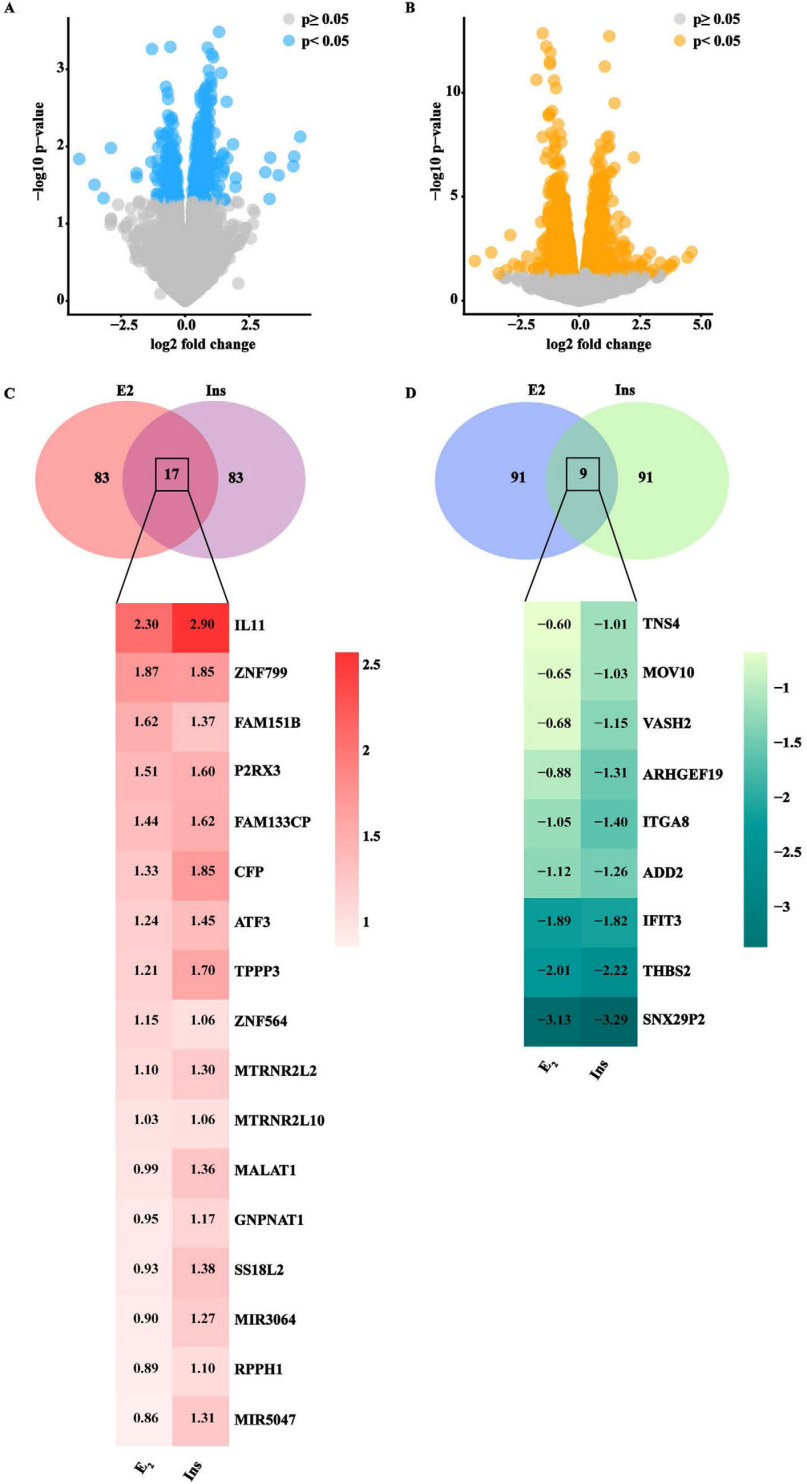
RNA‐seq analysis of BCAHC‐1 cells exposed to E2 and insulin. Volcano plots show differentially expressed genes in BCAHC‐1 cells treated for 12h with E2 (A) or insulin (B). *p* < 0.05 was set as a significant threshold. (C) Venn diagram and heat map depicting the genes shared among the first 100 up‐regulated genes by E2 or insulin (Ins). (D) Venn diagram and heat map depicting the genes shared among the first 100 down‐regulated genes by E2 or insulin (Ins). The log_2_ fold change of each gene is shown within the heat maps Abbreviations: E2, 17β‐estradiol.

**FIGURE 6:**
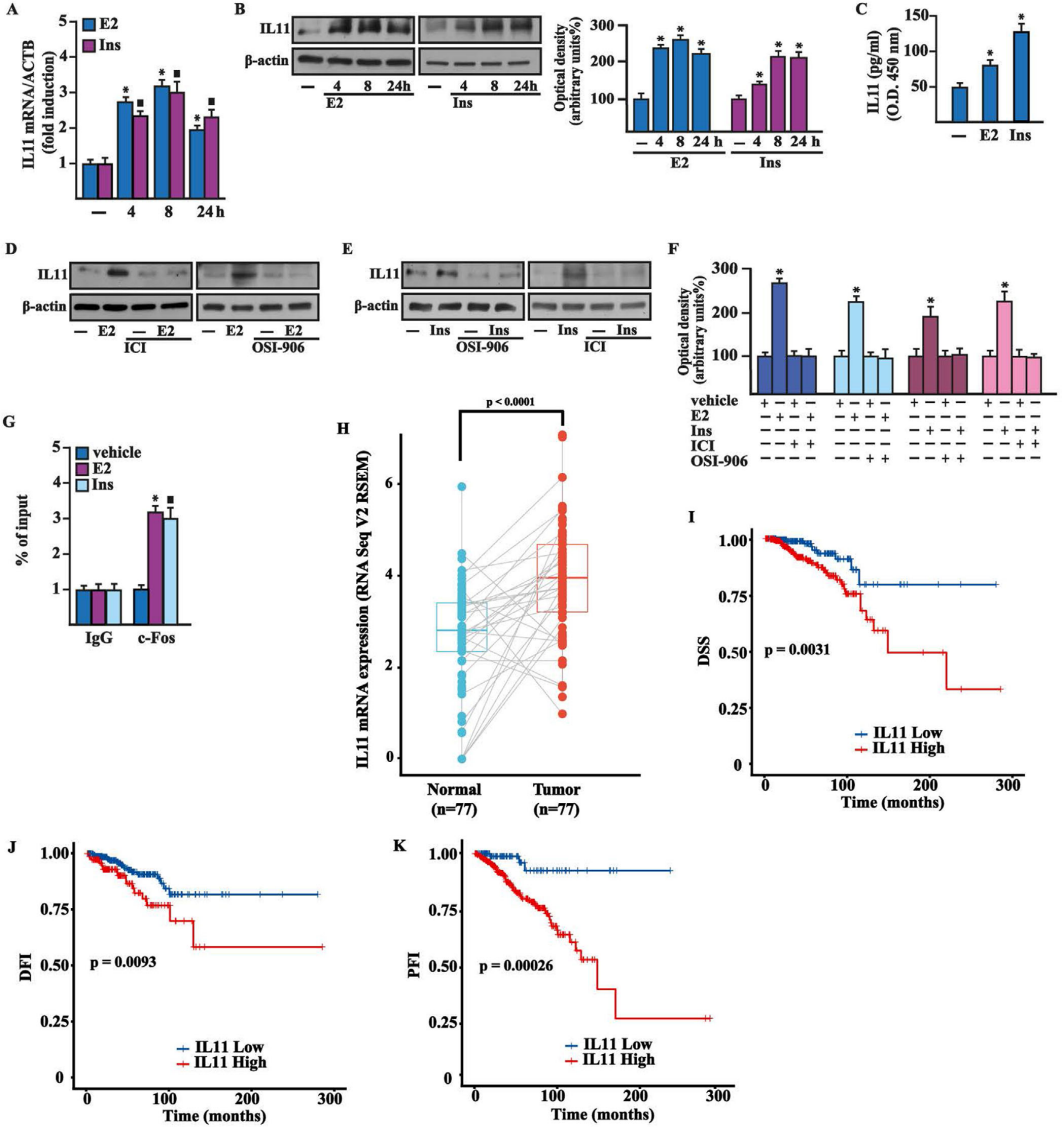
ERα46 and IR transduction pathways are both involved in the expression of IL11 induced by E2 and insulin in BCAHC‐1 cells. mRNA (A) and protein (B) expression of IL11 evaluated respectively by real‐time PCR and immunoblotting in BCAHC‐1 cells exposed to vehicle (−), 100 nM E2, or 10 nM insulin (Ins) for the indicated times. In RNA experiments, values are normalized to the actin beta (ACTB) expression and shown as fold changes of mRNA expression upon treatments relative to vehicle (−). Side panel shows densitometric analysis of the blots normalized to β‐actin. (C) IL11 levels evaluated by ELISA in the supernatants collected from BCAHC‐1 cells exposed to 100 nM E2 and 10 nM insulin (Ins) for 8 h. (D‐F) The upregulation of IL11 protein levels induced by 8 h treatment with 100 nM E2 (D) and 10 nM insulin (Ins) (E) is prevented in the presence of 1 μM ERα antagonist ICI and 1 μM IR inhibitor OSI‐906. (F) Densitometric analysis of the blots shown in panels D and E normalized to β‐actin. (G) Recruitment of c‐Fos to the AP‐1 sites located within the IL11 promoter sequence in BCAHC‐1 cells exposed to 100 nM E2 and 10 nM insulin (Ins) for 8 h. In control samples nonspecific IgG was used instead of the primary antibody. The amplified sequences were evaluated by real‐time PCR. Values represent the mean ± SD of three independent experiments performed in triplicate. (*) indicates *p* < 0.05 for cells exposed to treatments versus vehicle (−). (H) Pairwise comparison of IL11 expression of TCGA ER‐positive breast tumor samples and the adjacent normal tissues. (I‐K) Kaplan–Meier curves showing the correlation between IL11 expression and disease‐specific survival (DSS) (I), disease‐free interval (DFI) (J) and progression‐free interval (PFI) (K) in the TCGA cohort of ER‐positive BC patients Abbreviations: ERα46, 46 kDa ERα splice variant; E2, 17β‐estradiol; IR, insulin receptor; mRNA, messenger RNA.

Furthermore, we investigated the transcriptomic changes induced by E2 and insulin in BCAHC‐1 cells. High‐throughput RNA sequencing (RNA‐seq) analysis was performed. Library size‐normalized counts for the three samples (vehicle, E2, and insulin) were generated, and Figure 5 (panels A‐B) shows volcano plots depicting E2 and insulin differentially expressed genes (DEGs) relative to the vehicle. Based on the vast amount of the RNA‐seq data, we next assessed the first 100 upregulated and 100 down‐regulated DEGs after each treatment (Tables S2,S3). Focusing on DEGs modulated by both E2 and insulin, we found 17 upregulated (Figures 5C) and nine downregulated genes (Figure 5D). As the recognized cancer driver named IL11^28^ was the most induced gene shared by both treatments (Figure 5C), we aimed to provide further insights into its regulation in BCAHC‐1 cells. In this regard, we first ascertained that E2 and insulin induce IL11 mRNA (Figure 6A) and protein (Figure 6B) expression. Similarly, we found an increased secretion of IL11 in the medium of BCAHC‐1 cells upon treatment with E2 and insulin, as assessed by ELISA assays (Figure 6C). Remarkably, the silencing of ERα46 or the presence of ICI and OSI‐906 prevented IL11 expression prompted by E2 and insulin (Figures S2C and 6D,F), suggesting that ERα46 and IR regulate IL11 through a functional crosstalk. In addition, we found that c‐fos is recruited to the IL11 promoter region in BCAHC‐1 cells exposed to both E2 and insulin, as ascertained by chromatin immunoprecipitation assay (Figure 6G) . Afterward, to further appreciate the biological significance of IL11 in breast malignancy, we analyzed its expression in the TCGA cohort of ER‐positive BC samples. Notably, the pairwise comparison showed that IL11 levels are significantly higher in primary ER‐positive BC than in matched normal tissues (Figure 6H). We then sought to ascertain whether IL11 expression would predict the outcome of ER‐positive BC patients by evaluating disease‐specific survival, disease‐free interval, and progression‐free interval. Gene expression data were ranked according to low and high IL11 levels, and all possible points of separation and their significance were reported in the survivALL plots, which allowed the assessment of the most significant cut‐point (Figure S3). Overall, the relative Kaplan–Meier survival curves revealed worse outcomes in BC patients with high IL11 expression levels (Figure 6I,K).

### 3.5 IL11 induces pro‐tumorigenic genes and motility in CAFs

**FIGURE 7:**
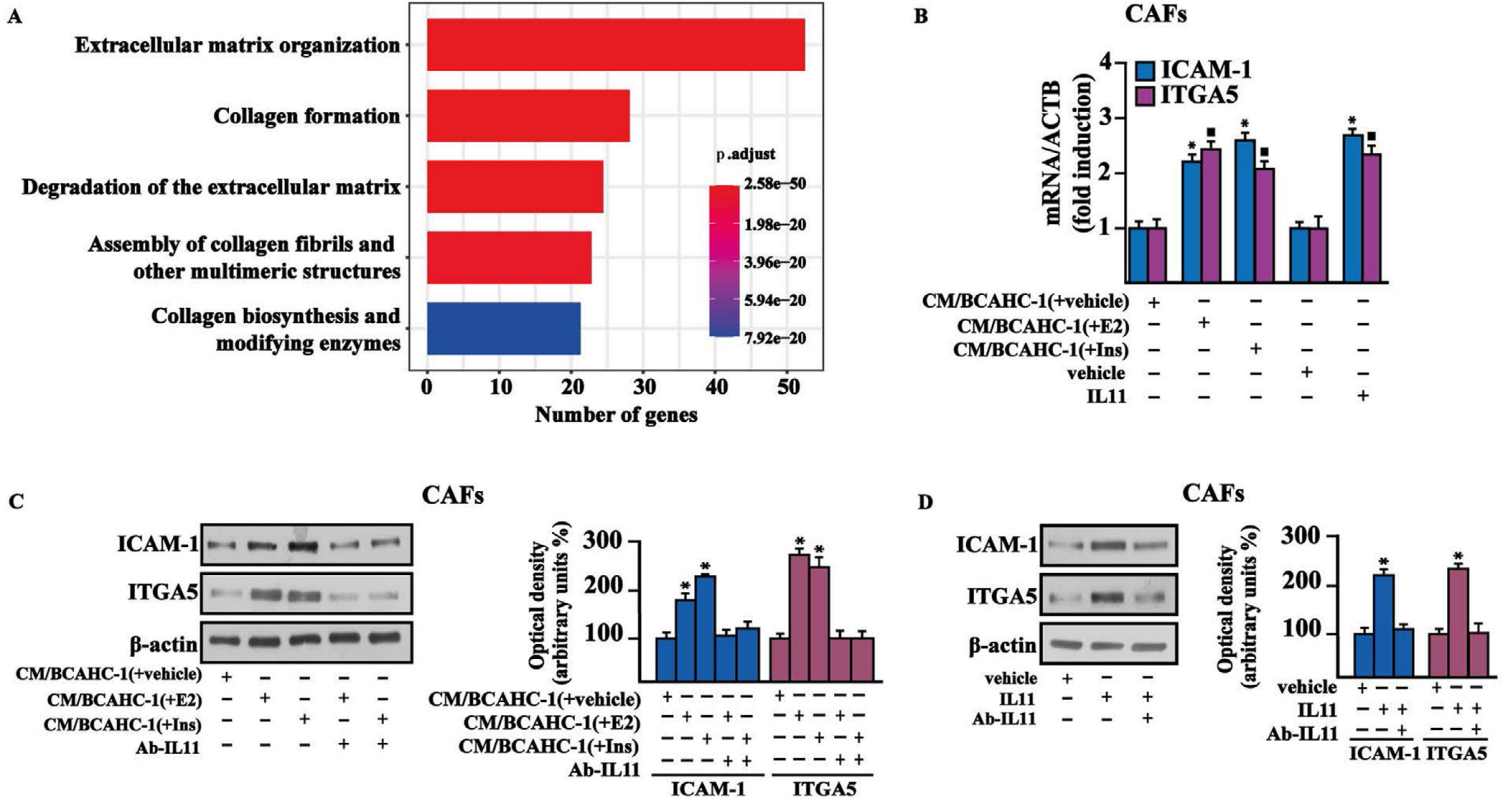
The paracrine action of IL11 promotes the expression of ICAM‐1 and ITGA5 in CAFs. (A) The bar plot shows the first five most significant IL11‐related pathways resulting from Reactome pathway analysis. The number of the genes included in each pathway is displayed along the x‐axis, while the different Reactome pathways are shown along the y‐axis. The color bar gradient indicates the range of significance (BH p‐adjusted values) of each pathway. (B) mRNA expression of ICAM‐1 and ITGA5 evaluated by real‐time PCR in CAFs exposed for 18 h to vehicle (−), 20 ng/ml IL11, or conditioned medium (CM) collected from BCAHC‐1 cells previously treated with vehicle, 100 nM E2 or 10 nM insulin (Ins). (C) Protein levels of ICAM‐1 and ITGA5 evaluated by immunoblotting in CAFs exposed for 18 h to conditioned medium (CM) collected from BCAHC‐1 cells previously treated with vehicle, 100 nM E2, or 10 nM insulin (Ins), in the presence or absence of 200 ng/ml IL11 neutralizing‐antibody (Ab‐IL11). (D) Protein levels of ICAM‐1 and ITGA5 in CAFs exposed to vehicle or 20 ng/ml IL11 for 18 h in the presence or absence of 200 ng/ml Ab‐IL11. Side panels show densitometric analysis of the blots normalized to β‐actin. * indicates *p* < 0.05 for cells exposed to treatments versus vehicle (−) Abbreviations: CAF, cancer‐associated fibroblasts; IL11, interleukin 11; PCR, polymerase chain reaction.

**FIGURE 8:**
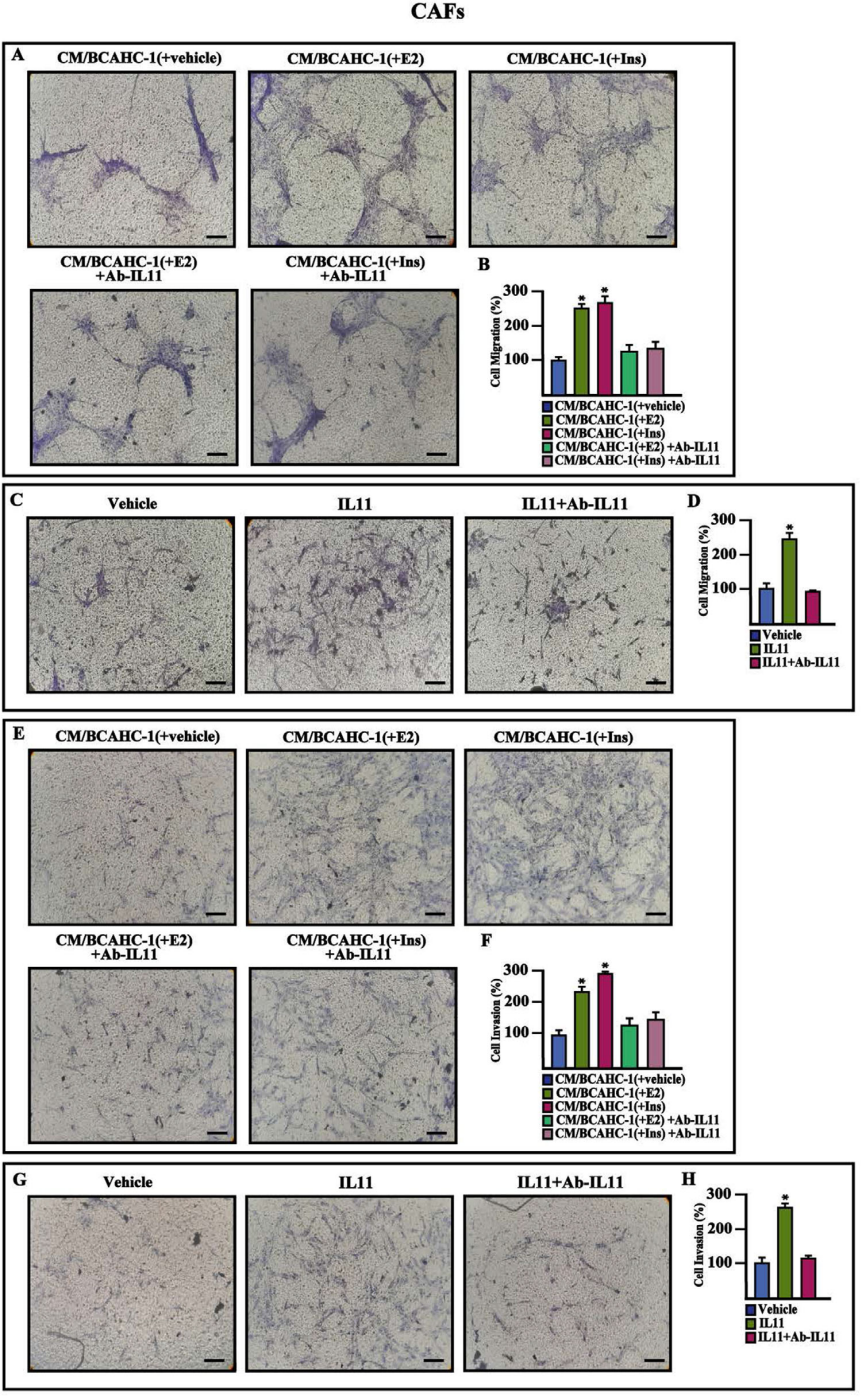
BCAHC‐1 cells‐derived IL11 induces migration and invasion in CAFs. (A‐D) Transwell migration assay in CAFs exposed for 18 h to conditioned medium (CM) collected from BCAHC‐1 cells previously treated with vehicle, 100 nM E2, and 10 nM insulin (Ins) (A), or exposed for 18 h to vehicle or 20 ng/ml IL11 (C) in the presence or absence of 200 ng/ml Ab‐IL11. (E‐H) Transwell Matrigel invasion assay in CAFs exposed for 18 h to conditioned medium (CM) collected from BCAHC‐1 cells previously treated with vehicle, 100 nM E2, and 10 nM insulin (Ins) (E), or exposed for 18 h to vehicle or 20 ng/ml IL11 (G) in the presence or absence of 200 ng/ml Ab‐IL11. Panels B, D, F, and H show the number of cells counted in at least 10 random fields in three independent experiments performed in triplicate. Scale bar: 200 μM. Values represent the mean ± SD of three independent experiments performed in triplicate. * indicates *p* < 0.05 for cells exposed to treatments versus vehicle Abbreviations: CAF, cancer‐associated fibroblasts; IL11, interleukin 11; SD, standard deviation.

We further performed Reactome pathway enrichment analysis using the top 1000 IL11‐correlated genes, ranked by Pearson correlation coefficient and clustered in transduction pathways. The five most significantly enriched pathways related to IL11 shown in the bar plot of Figure 7 (panel A) suggest that IL11 expression in ER‐positive BC is associated with pro‐invasive genes. Based on the aforementioned results, we aimed to provide novel mechanistic insights into the paracrine action of IL11 on main components of the BC microenvironment such as CAFs, which express the IL11 receptor, IL11Rα (Interleukin‐11 receptor alpha), and the glycoprotein 130 (gp130) co‐receptor (data not shown). In particular, we focused on IL11 capacity to regulate the two pro‐tumorigenic genes, intercellular adhesion molecule 1 (ICAM‐1) and integrin alpha 5 (ITGA5), which belong to the most enriched IL11‐related pathway, namely “extracellular matrix (ECM) organization,” as indicated above (Figure 7A). In CAFs cultured in conditioned medium (CM) collected from BCAHC‐1 cells previously exposed to E2 or insulin and in CAFs treated with IL11, we found an increased expression of ICAM‐1 and ITGA5 at both mRNA (Figure 7B) and protein (Figure 7C,D) levels. Notably, immunoblots experiments showed that these stimulatory effects on ICAM‐1 and ITGA5 of IL11 are abolished using the neutralizing IL11 antibody (Ab‐IL11) (Figure 7C,D). Similarly, the migratory and invasive properties of CAFs, either cultured with CM collected from BCAHC‐1 cells upon E2 and insulin exposure or treated with IL11, were inhibited by the neutralizing IL11 antibody (Figure 8A,H). Taken together, these results suggest that E2 and insulin stimulate the secretion of IL11 by BCAHC‐1 cells for its paracrine action on CAFs, hence corroborating the functional interaction between BC cells and the surrounding microenvironment.

## 4 DISCUSSION

The molecular characterization of BC has helped identify four different intrinsic subtypes: luminal A and luminal B (expressing ER and/or PR) and human epidermal growth factor receptor 2 (HER2)‐enriched and basal‐like, which expresses certain markers of basal cellular origin, but not ER, PR, and HER2.^29^ Integrated analysis of gene expression profiling from large BC cohorts, along with next‐generation sequencing studies, has revealed that each of the four BC subtypes exhibits molecular heterogeneity, specific protein expression patterns, and unique activated transduction pathways.^2^ These biological features may influence therapeutic responses and clinical outcomes.^3^ For instance, approximately 40% of ERα‐positive BC patients fail to respond to endocrine manipulation.^30^ The identification of different ERα splicing variants lacking one or more exons has complicated the features of BC.^31^ In this regard, antibodies used in immunohistochemistry are usually directed against epitopes located in the ERα C‐terminal region.^32^ Consequently, the expression of ERα splicing variants that may trigger differential therapeutic outcomes cannot be ruled out. As other antibodies recognize epitopes encoded by the first exon of the ERα gene,^32^ certain ERα splice variants might not be detected, thus complicating the evaluation of ERα expression in BC patients. For instance, ERα46 lacks the first 173 amino acids of the N‐terminal region of the full‐length protein known as ERα66. Alternative splicing of exon 1E directly to exon 2 of the ERα gene has been proposed as the main mechanism underlying the generation of ERα46, although further events might account for its expression, including proteolytic processes^9^, ^33^, ^34^. ERα46 is frequently expressed in human BCs, and the ERα46/ERα66 expression ratio is negatively correlated with tumor grade.^35^ Accordingly, the overexpression of ERα46 in MCF‐7 cells antagonizes the stimulatory action mediated by ERα66, thus inhibiting AF‐1‐sensitive estrogen target genes and cell cycle arrest.^11^ In addition to its genomic function, ERα46 acting as a plasma membrane‐associated form of ERα, activates the c‐Src‐PI3K/Akt transduction pathway in vascular endothelium.^10^ In this context, BCAHC‐1 cells represent a novel model system for the comprehensive evaluation of the role of ERα46 in BCs lacking ERα66. For instance, in these cells, E2 could not transactivate ERα46 to modulate its protein levels and induce its nuclear translocation or to regulate classical estrogen target genes; however, ERα46 was required for certain transcriptional changes and growth events prompted by E2.

IR‐A and IR‐B isoforms are involved in pathophysiological processes triggered by insulin, including development, differentiation, metabolism as well as obesity, diabetes, and cancer.^36^, ^37^ For instance, activated IR has been associated with mitogenic signaling pathways and poor prognosis in BC patients.^38^ Besides, elevated IR‐A levels and a high IR‐A/IR‐B ratio correlate with BC aggressiveness and resistance to therapeutics.^36^ Indeed, overexpression of IR‐A has been found in BC cells, which partly explains the association between increased BC risk and altered insulin levels in both obese and type 2 diabetic patients.^38^ Remarkably, a recent epidemiological analysis, which was based on 313907 diabetic patients from the United Kingdom, showed that the transition from vascular illnesses to cancer was the leading contributor to diabetes‐related death.^39^ As the primary BCAHC‐1 BC cells are characterized by ERα46 expression together with a predominant expression of IR‐A, this model system represents a unique and promising opportunity to further dissect the previously reported role of estrogen and insulin in BC progression.^14^, ^15^, ^16^

In this study, we aimed to provide novel insights into both E2/ERα46 and insulin/IR transduction pathways. In accordance with previous data revealing that a cooperative interaction between ERα66 and IR transduction pathways plays a critical role in breast carcinogenesis, tumor cell proliferation, differentiation and survival,^40^, ^41^, ^42^ we demonstrated that regulatory crosstalk occurs between ERα46 and IR upon E2 and insulin stimulation for BCAHC‐1 cell growth and pulmonary metastasis. In particular, we ascertained that ERα46 and IR signaling might trigger these responses through the induction of c‐Fos and Cyclin D1. Moreover, we showed that the functional cooperation between ERα46 and IR might also rely on their physical interaction, in addition to other mechanisms triggering BC metastasis.^14^ Next, we used a high‐throughput RNA‐seq approach to comprehensively identify the transcriptional changes induced by estrogen and insulin in BCAHC‐1 cells. An IL‐6 family member, namely IL11, emerged as the most upregulated gene by both E2 and insulin. IL‐6 family cytokines, which play relevant roles in regulating immunity, hematopoiesis, development, and metabolism,^43^ are also involved in inflammation, autoimmunity, and cancer progression.^43^, ^44^, ^45^ Regarding IL11, previous studies have found that its expression is higher in BC relative to normal breast tissues and correlates with poor disease outcomes.^46^ Besides, the role of IL11 in bone metastasis of BCs has been assessed.^47^ In this study, after demonstrating that both ERα46 and IR transduction cascades stimulated by E2 and insulin in BCAHC‐1 cells are involved in the induction of IL11, we performed an integrated bioinformatics analysis in ER‐positive BC datasets. In this regard, we assessed that the mRNA expression of IL11 is upregulated in ER‐positive BC respect to paired normal breast tissues and is associated with worse survival rates. Nicely supporting these findings, we found that several cell invasion and metastasis pathways are associated with IL11 expression. The “ECM organization” pathway, which has been involved in BC progression, includes numerous proteins involved in ECM remodeling and cell‐to‐cell and cell‐to‐matrix interactions like ICAM‐1 and ITGA5.^48^, ^49^, ^50^, ^51^, ^52^ Notably, the “ECM organization” pathway was significantly correlated with IL11. Accordingly, both ICAM‐1 and ITGA5 were regulated in CAFs by either IL11 secreted by BCAHC‐1 cells upon E2 and insulin exposure or recombinant IL11 protein, as demonstrated using the neutralizing antibody anti‐IL11. Furthermore, the presence of the neutralizing antibody anti‐IL11 inhibited the invasive and migratory properties of CAFs. Hence, these data point out for the first time the role of IL11 produced by BC cells in stimulating the aggressive features of the tumor microenvironment components such as CAFs. Further studies are warranted for additional evaluation of the estrogen and insulin action leading to a microenvironmental phenotype that facilitates BC metastasis through the involvement of IL11.

## 5 CONCLUSION

**FIGURE 9:**
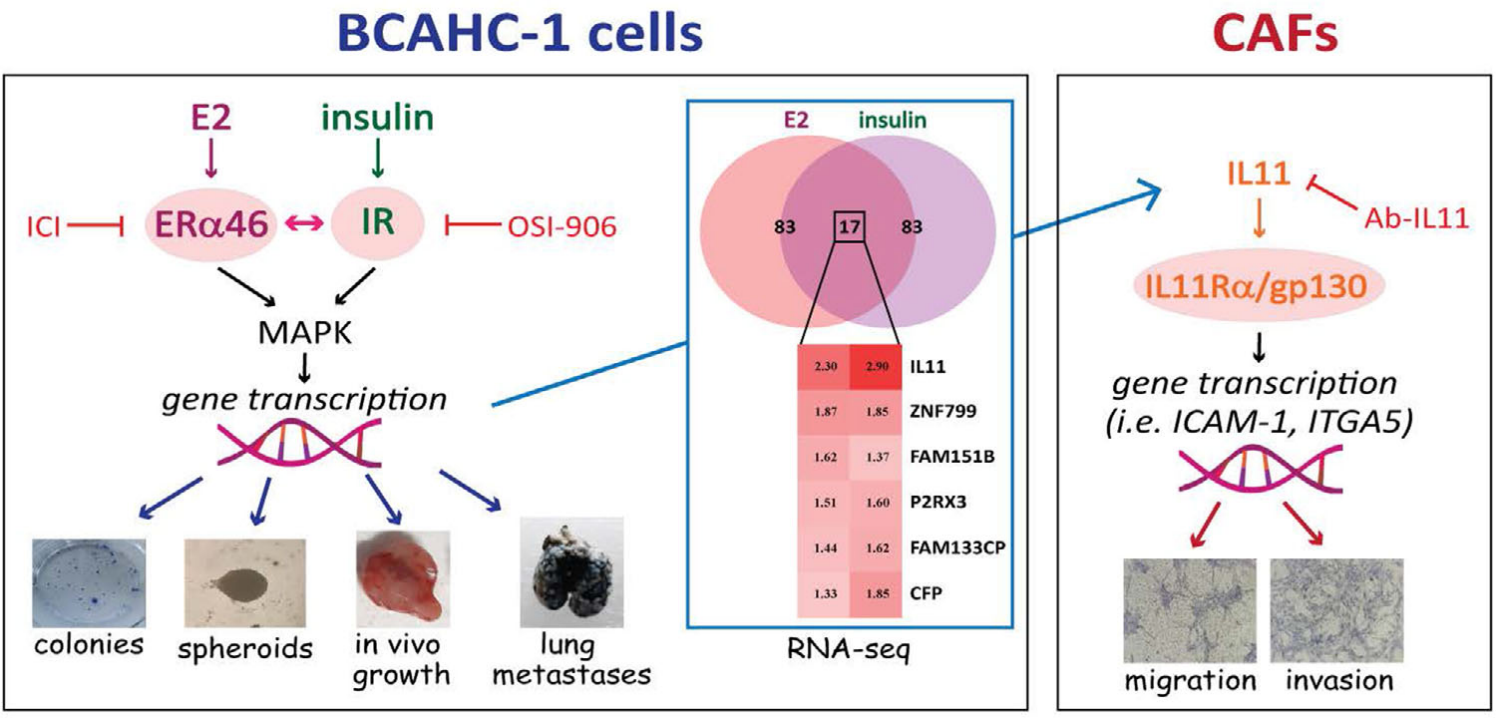
Cartoon depicting the cooperation between the ERα46 and IR transduction pathways leading to the growth and metastasis of BCAHC‐1 cells as well as stimulatory effects within the tumor microenvironment

The unique receptor expression profile and the biological responses of BCAHC‐1 cells provide a valuable resource to inform the molecular mechanisms triggering the progression of certain BC types. For instance, BCAHC‐1 cells may represent a unique model system to demonstrate the role of ERα46 and IR either alone or through a functional liaison for crucial interactions within the tumor microenvironment, leading to BC metastasis (as schematically depicted in Figure 9). Similarly, our findings open a new avenue regarding the usefulness of ERα46 and IR in setting suitable therapeutic options for pathophysiological conditions characterized by dysregulated levels of ligands of these receptors. Nevertheless, whether and to what extent the findings obtained in BCAHC‐1 cells may be important for BC remain to be determined in next studies.

## CONFLICT OF INTEREST

The authors declare no conflict of interest.

## ETHICS APPROVAL AND CONSENT TO PARTICIPATE

Informed written consent was obtained from the patients according to the Institutional Review Board (IRB) of Annunziata Hospital (Cosenza, Italy). The animal research project was approved by the Italian Ministry of Health, Rome (authorization n. 706/2018‐PR). All experimental procedures were approved by the Bioethics Committee on Animal Experiments (OPBA) of the Department of Biology, Ecology and Earth Sciences, University of Calabria.

## FUNDING INFORMATION

Fondazione AIRC supported Ernestina Marianna De Francesco (Start‐Up Grant 21651), Antonino Belfiore (IG n. 23369) and Marcello Maggiolini (IG n. 21322). Damiano Cosimo Rigiracciolo was supported by Italian Minister of University and Research (MIUR, D.D. n. 3407/2018)‐PON R&I 2014–2020 ‘AIM Attrazione e Mobilità Internazionale’.

## AUTHOR CONTRIBUTIONS

Francesca Cirillo, Marcello Maggiolini, Rosamaria Lappano and Rita Guzzi conceived and designed the study. Francesca Cirillo, Michele Pellegrino, Marianna Talia, Ida Daniela Perrotta, Damiano Cosimo Rigiracciolo, Asia Spinelli, Domenica Scordamaglia, and Lucia Muglia performed the experiments. Marianna Talia, Rita Guzzi, Ernestina Marianna De Francesco, and Antonino Belfiore contributed to data analysis. Anna Maria Miglietta provided clinical samples. Marcello Maggiolini and Rosamaria Lappano wrote the manuscript, supervised the research, and interpreted results. Ernestina Marianna De Francesco, Antonino Belfiore and Marcello Maggiolini secured funding.

## Supporting information

Supplementary Fig. 1

Supplementary Fig. 2

Supplementary Fig. 3

Supplementary Materials and Methods

Supplementary TableS1

Supplementary TableS2

Supplementary TableS3
