# Supplementary material for: Estrogen receptor variant ERα46 and insulin receptor drive in primary breast cancer cells growth effects and interleukin 11 induction prompting the motility of cancer‐associated fibroblasts
Source: Clin Transl Med. 2021 Nov 4;11(11):e516. doi: 10.1002/ctm2.516 (PMC8567034; doi:10.1002/ctm2.516)
Supplement: Supplementary file 1 — Supplementary Fig. 1 [file CTM2-11-e516-s001.pdf]

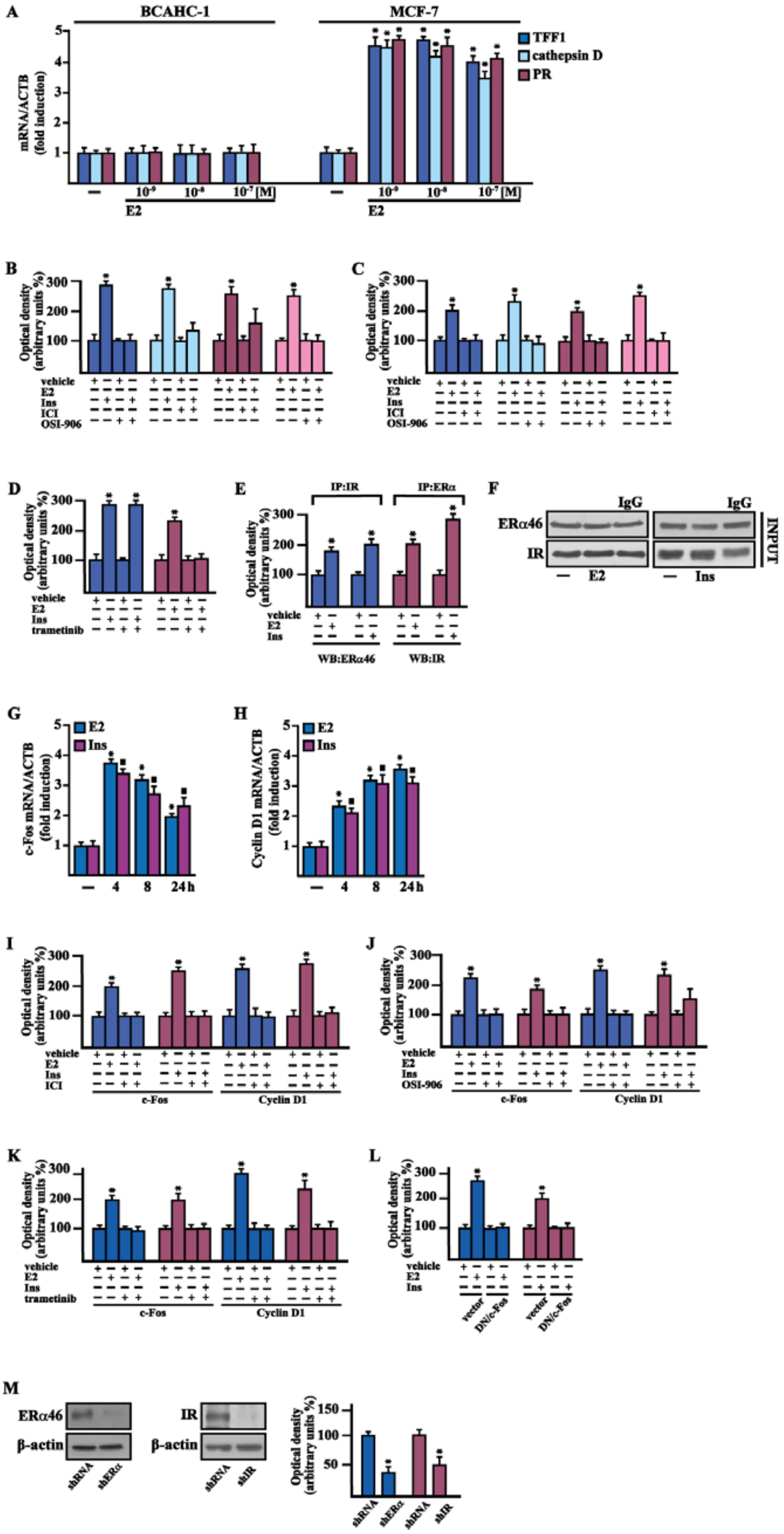

A
BCAHC-1
MCF-7
TFF1
cathepsin D
PR
mRNA/ACTB (fold induction)
E2
B
C
D
E
F
IP:IR
IP:ERα
WB:ERα46
WB:IR
ERα46
IR
IgG
INPUT
Ins
G
H
c-Fos mRNA/ACTB (fold induction)
Cyclin D1 mRNA/ACTB (fold induction)
I
J
K
L
M
Optical density (arbitrary units %)
vehicle
ICI
OSI-906
trametinib
c-Fos
Cyclin D1
vector
DN/c-Fos
β-actin
shRNA
shERα
shIR

Supplementary Fig. 1. (A) mRNA expression of TFF1, cathepsin D and PR in BCAHC-1 and MCF-7 cells treated for 24 h with vehicle (−) and the indicated concentrations of E2, as evaluated by real-time PCR. Values are normalized to the actin beta (ACTB) expression and shown as fold changes of mRNA expression induced by treatments compared to vehicle (−). (B) Densitometric analysis of the blots shown in panels A-D of figure 3 normalized to IR, which served as loading control. (C) Densitometric analysis of the blots shown in panels E-H of figure 3 normalized to ERK2, which served as loading control. (D) Densitometric analysis of the blots shown in panels I-J of figure 3 normalized to IR, which served as loading control. (E) Densitometric analysis of the blots shown in panels K-L of figure 3, which were normalized to IR or ERα, as indicated. (F) Total lysates (input), in reference to co-immunoprecipitation assays shown in panels K-L of figure 3, were evaluated as control. In control samples, nonspecific IgG was used instead of the primary antibody. (G-H) mRNA expression of c-Fos (G) and Cyclin D1 (H) in BCAHC-1 cells treated with vehicle (−) and 100nM E2 or 10nM insulin (Ins) for the indicated times, as evaluated by real-time PCR. Values are normalized to the actin beta (ACTB) expression and shown as fold changes of mRNA expression induced by treatments compared to vehicle (−). (I) Densitometric analysis of the blots shown respectively in panels M-N of figure 3, normalized to β-actin. (J) Densitometric analysis of the blots shown respectively in panels O-P of figure 3, normalized to β-actin. (K) Densitometric analysis of the blots shown respectively in panels Q-R of figure 3, normalized to β-actin. (L) Densitometric analysis of the blots shown in panels S-T of figure 3 normalized to β-actin. (M) Representative immunoblots of the efficacy of ERα or IR silencing. Side panel shows densitometric analysis of the blots normalized to β-actin. Values represent the mean ± SD of three independent experiments performed in triplicate. (*), (■) indicate $p < 0.05$ for cells exposed to treatments versus vehicle (−).
