# Supplementary material for: Estrogen receptor variant ERα46 and insulin receptor drive in primary breast cancer cells growth effects and interleukin 11 induction prompting the motility of cancer‐associated fibroblasts
Source: Clin Transl Med. 2021 Nov 4;11(11):e516. doi: 10.1002/ctm2.516 (PMC8567034; doi:10.1002/ctm2.516)
Supplement: Supplementary file 2 — Supplementary Fig. 2 [file CTM2-11-e516-s007.pdf]

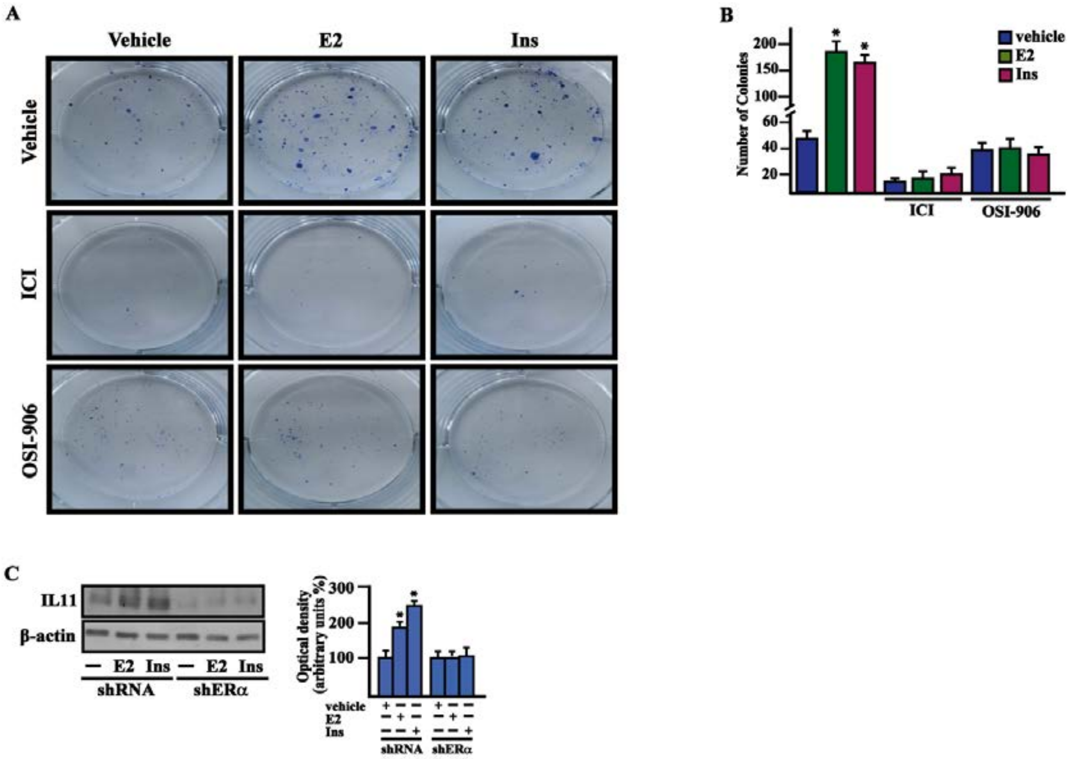

Supplementary Fig. 2. (A) Colony formation assay in BCAHC-1 cells exposed to vehicle, 100nM E2 or 10nM insulin (Ins) alone and in combination with 1µM ERα antagonist ICI 182,780 (ICI) and 1µM IR inhibitor OSI-906. (B) After 10 days of treatment, the plates were stained with Giemsa, and colonies were counted. (C) The up-regulation of IL11 protein levels induced by 8 h treatment with 100nM E2 and 10nM insulin (Ins) is prevented silencing ERα46 expression. Side panel shows densitometric analysis of the blot normalized to β-actin. Representative immunoblot of the efficacy of ERα silencing is shown in supplementary figure 1 (panel M). Values represent the mean ± SD of three independent experiments performed in triplicate. (*) indicates $p < 0.05$ for cells exposed to treatments versus vehicle (−).
