# Supplementary material for: Estrogen receptor variant ERα46 and insulin receptor drive in primary breast cancer cells growth effects and interleukin 11 induction prompting the motility of cancer‐associated fibroblasts
Source: Clin Transl Med. 2021 Nov 4;11(11):e516. doi: 10.1002/ctm2.516 (PMC8567034; doi:10.1002/ctm2.516)
Supplement: Supplementary file 3 — Supplementary Fig. 3 [file CTM2-11-e516-s004.pdf]

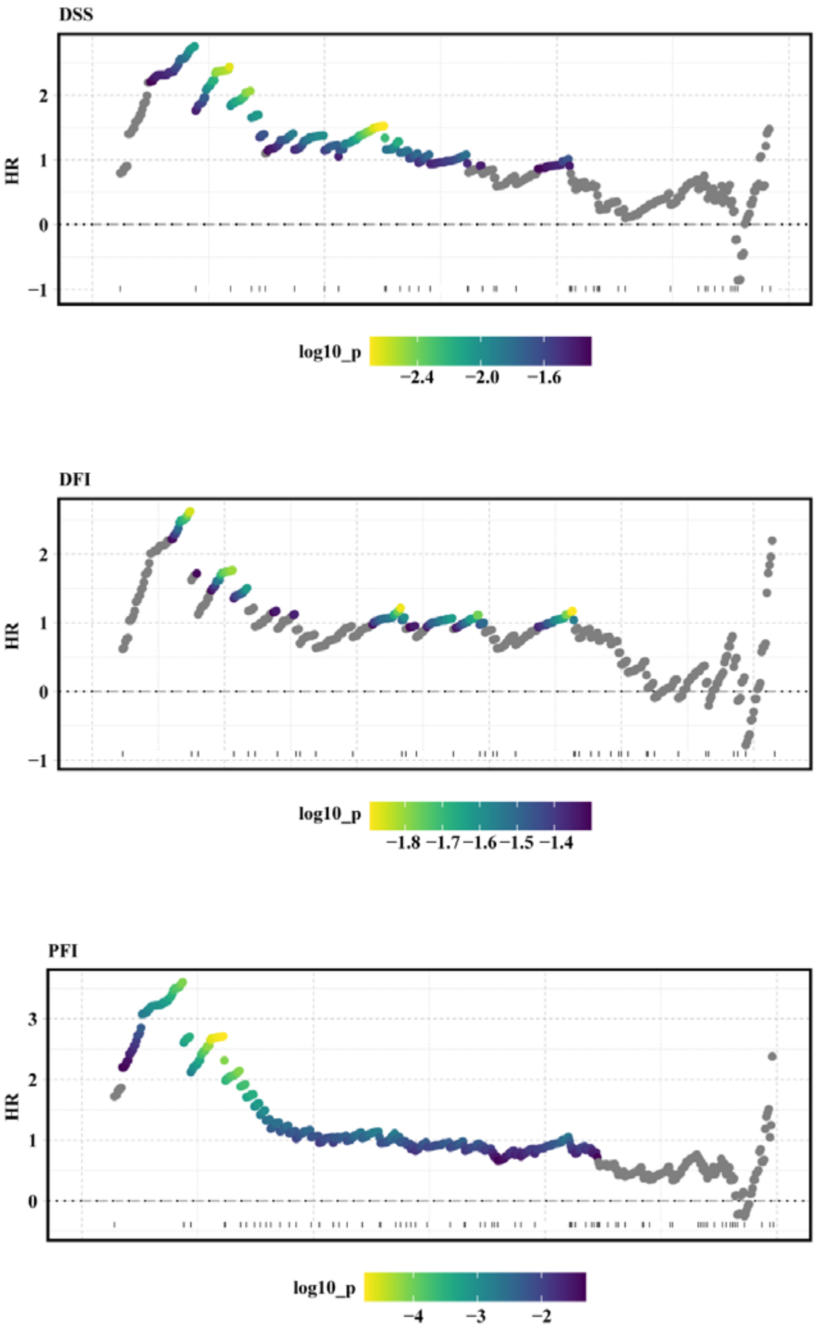

Supplementary Fig. 3. The plotALL function of survivALL calculating hazard ratios (HR) (y-axis) for all possible IL11 cut-points to be examined. TCGA breast cancer patients ordered by increasing expression of IL11 (x-axis). The color bar gradient stands for range of the most significant points-of-separation of the population (low-high significance = blue-yellow gradient) based on IL11 expression and disease specific survival (DSS), disease-free interval (DFI) and progression-free interval (PFI) of each patient.
