# Supplementary material for: Estrogen receptor variant ERα46 and insulin receptor drive in primary breast cancer cells growth effects and interleukin 11 induction prompting the motility of cancer‐associated fibroblasts
Source: Clin Transl Med. 2021 Nov 4;11(11):e516. doi: 10.1002/ctm2.516 (PMC8567034; doi:10.1002/ctm2.516)
Supplement: Supplementary file 4 — Supplementary Materials and Methods [file CTM2-11-e516-s003.pdf]

Supplementary Materials and Methods

Reagents. The proteasome inhibitor MG132 and the DNA-hypomethylating agent 5-aza-2′-deoxycytidine (5-aza), 17β-Estradiol (E2), Insulin (Ins) and the ERα antagonist ICI-182780 (ICI) were purchased from Merck Life Science (Milan, Italy). The MEK inhibitor trametinib and the insulin receptor (IR) inhibitor OSI-906 were obtained from MedChemExpress (DBA, Milan, Italy). Recombinant human IL11 protein was purchased from Abcam (DBA, Milan, Italy) and solubilized in PBS with 1% BSA. Anti-IL11 neutralizing antibody (MAB218) was purchased from R&D Systems (BioTechne, Milano, Italy). All compounds were dissolved in DMSO, except insulin that was solubilized in water.

TRAPeze assay. Telomerase activity of BCAHC-1 cells were determined using the TRAPeze RT Telomerase Detection kit S7710 (Millipore, Merck, Milan, Italy) following instructions by the manufacturer. In brief, cells at 70−80% confluence were washed in PBS and lysed at 4°C in CHAPS lysis buffer. Cell debris was removed by centrifugation at 13,000 rpm for 20 min at 4°C and protein was quantified using BCA protein assay according to the manufacturer's instructions (Thermo Fisher Scientific, Milan, Italy). A total of 1 μg protein was used for TRAPeze assay and telomerase activity was quantified using platform Quant Studio7 Flex Real-Time PCR System (Thermo Fisher Scientific, Milan, Italy).

Flow cytometry analysis. BCAHC-1 cells (2×10^6) were fixed in ice-cold methanol for 10 min, permeabilized in 0.1% Triton X-100 in PBS for 15 min and incubated with primary antibodies anti-cytokeratin-FITC (Beckman-Coulter, Milan, Italy) and anti-FAPα (Santa Cruz Biotechnology, DBA, Milan, Italy) for 1 h at 4 °C. Alexa Fluor 594-conjugated secondary antibody (Thermo Fisher Scientific, Milan, Italy) was subsequently applied for another 30 min. Samples were then analyzed with CytoFLEX flow cytometry (Beckman-Coulter, Milan, Italy).

Library preparation and sequencing. Total RNA from BCAHC-1 cells was extracted with RNeasy mini kit according to manufacturer's instructions (Qiagen, Bioset, Catanzaro, Italy). TruSeq Stranded

mRNA kit (Illumina, San Diego, CA) has been used for library preparation following the manufacturer's instructions (library type: fr-firstrand). RNA samples were quantified and quality tested by Agilent 2100 Bioanalyzer RNA assay (Agilent technologies, Santa Clara, CA) or by Caliper (PerkinElmer, Waltham, MA). Final libraries were check with both Qubit 2.0 Fluorometer (Invitrogen, Carlsbad, CA) and Agilent Bioanalyzer DNA assay or Caliper (PerkinElmer, Waltham, MA). Libraries were then prepared for sequencing and sequenced on single-end 75 bp mode on NextSeq 500 (Illumina, San Diego, CA).

RNA-Seq bioinformatics analysis. Raw data was processed for both format conversion and de-multiplexing by Bcl2Fastq 2.20 version of the Illumina pipeline. Adapter sequences were masked with Cutadapt v1.11 from raw fastq data using the following parameters: --anywhere (on both adapter sequences) --overlap 5 --times 2 --minimum-length 35 --mask-adapter. Lower quality bases and adapters were then removed by ERNE (1) software. Next, the reads were aligned on reference hg38 genome/transcriptome with STAR (2) (default parameters), a splice junction mapper for RNA-Seq reads. It aligns RNA-Seq reads to genomes and then analyzes the mapping results to identify splice junctions between exons. The full-length transcripts representing multiple spliced variants for each gene locus were assembled and quantified by Stringtie (3) (default parameters). The RSeqQC (3) package was used to perform quality control.

A pairwise differential expression analysis was assessed. htseq-count (4) was used to preprocess RNA-Seq data for differential expression analysis by counting the overlap of reads with genes. DESeq2 (5) was then used to perform comparisons between expression levels of genes and transcripts by fitting a Generalized Linear Model (GLM) for each gene in order to improve stability and interpretability of estimates. Normalization was performed using the median-of-ratios method (6); statistical significance was determined using a Wald test (5).

Gene expression studies. Total RNA was extracted, and cDNA was synthesized by reverse transcription as previously described (7). The expression of selected genes was quantified by real-

time PCR using platform Quant Studio7 Flex Real-Time PCR System (Thermo Fisher Scientific, Milan, Italy). Gene-specific primers were designed using Primer Express version 2.0 software (Applied Biosystems) and are as follows: 5’-GCCCCCCGTGAAAGAC-3’ (TFF1 forward) and 5’-CGTCGAAACAGCAGCCCTTA-3’ (TFF1 reverse); 5′-CTGGATCCACCACAAGTACAACA-3′ (cathepsin D forward) and 5′-CGAGCCATAGTGGATGTCAAAC-3′ (cathepsin D reverse); 5’-GAGTTGTGAGAGCACTGGATGCT-3’ (PR forward) and 5’-CAACTGTATGTCTTGACCTGGTGAA-3’ (PR reverse); 5′-CGAGCCCTTTGATGACTTCCT-3′ (c-Fos forward) and 5′-GGAGCGGGCTGTCTCAGA-3′ (c-Fos reverse); 5′-AGCTGTGCATCTACACCGAC-3′ (cyclin D1 forward) and 5′-GAAATCGTGCGGGGTCATTG-3′ (cyclin D1 reverse); 5′-ATGAACTGTGTTTGCCGCCTG-3′ (IL11 forward) and 5′-GTCTGGGGAAACTCGAGGG-3′ (IL11 reverse); 5′-TCCGTGAAGCTGTGTTGTCC-3′ (IL11RA forward) and 5′-GTTCATGCCCTAGCCCAGAG-3′ (IL11RA reverse); 5′-CCCTGTATCACAGACTGGCAA-3′ (gp130 forward) and 5′-TCCTTTGGAAGGTGCTCTTAAAT-3′ (gp130 reverse); 5′-AGCTTCGTGTCCTGTATGGC-3′ (ICAM-1 forward) and 5′-TTTTCTGGCCACGTCCAGTT-3′ (ICAM-1 reverse); 5′-GGCTCCTTCTTCGGATTCTCA-3′ (ITGA5 forward) and 5′-GGCTGGCTGGTATTAGCCTT-3′ (ITGA5 reverse); 5′-AAGCCACCCCACTTCTCTCTAA-3′ (ACTB forward) and 5′-CACC TCCCCTGTGTGGACTT-3′ (ACTB reverse). Assays were performed in triplicate and the results were normalized for actin beta (ACTB) expression and then calculated as fold induction of RNA expression.

The standard curves for the mRNA levels of ESR1 (ERα), IGF1R, INSR A (IR-A), INSR B (IR-B) and ACTB (used as an internal control) were generated using serially diluted solutions (1-10^4-fold) of standard cDNA derived from MCF-7 cells as template, for the mRNA levels of AR and ESR2 (ERβ) from LNCaP cells, for the mRNA levels of EGFR, ERBB2, ERBB3, GPER, GR and MR from SkBr3 cells, for the mRNA levels of PGRA and PGRB from T47D cells. Gene-specific primers are

as follows: 5′-AGAGGGCATGGTGGAGATCTT-3′ (ESR1 C-E domains forward) and 5′-CAAACTCCTCTCCCTGCAGATT-3′ (ESR1 C-E domains reverse); 5′-AATTCAGATAATCGACGCCAG-3′ (ESR1 A-B domains forward) and 5′-GTGTTTCAACATTCTCCCTCCTC-3′ (ESR1 A-B domains reverse); 5′-TGGTGGAGAACGACCATATCC-3′ (IGF1R forward) and 5′-CGATTAACTGAGAAGAGGAGTTCGA-3′ (IGF1R reverse); 5′-CTGGTCTCCACCATTCGAGT-3′ (IR-A forward) and 5′-CGAGATGGCCTGGGGACG-3′ (IR-A reverse); 5′-CTGGTCTCCACCATTCGAGT-3′ (IR-B forward) and 5′-CCTCGGCACCAGTGCCTG-3′ (IR-B reverse); 5′-AGGATGCTCTACTTCGCCCC-3′ (AR forward) and 5′-ACTGGCTGTACATCCGGGAC-3′ (AR reverse); 5′-GACCACAAGCCCAAATGTGTT-3′ (ESR2 forward) and 5′-AACTGGCGATGGACCACTAAA-3′ (ESR2 reverse); 5′-CCCTCCTGAGCTCTCTGAGT-3′ (EGFR forward) and 5′-GTTTCCCCCTCTGGAGATGC-3′ (EGFR reverse); 5′-CACCTACAACACAGACACGTTTGA-3′ (ERBB2 forward) and 5′-TCCCACGTCCGTAGAAAGGTA-3′ (ERBB2 reverse); 5′-CTACCACCACTCTTTGAACTGGACCAAGG-3′ (ERBB3 forward) and 5′-TCTATGCTCTCACCCCGTTCCAAGTATCG-3′ (ERBB3 reverse); 5′-ACACACCTGGGTGGACACAA-3′ (GPER forward) and 5′-GGAGCCAGAAGCCACATCTG-3′ (GPER reverse); 5′-GAACTGGCAGCGGTTTTATC-3′ (GR forward) and 5′-TCTCGGGGAATTCAATACTCA-3′ (GR reverse); 5′-GCTTTGATGGTAACTGTGAAGG-3′ (MR forward) and 5′-TGTGTTGCCCTTCCACTGCT-3′ (MR reverse); 5′-CCTCGGACACCTTGCCTGAA-3′ (PGRA forward) and 5′-CGCCAACAGAGTGTCCAAGAC-3′ (PGRA reverse); 5′-CCTGAAGTTTCGGCCATACCT-3′ (PGRB forward) and 5′-AGCAGTCCGCTGTCCTTTTCT-3′ (PGRB reverse).

Reporter gene assays and gene silencing experiments. The firefly luciferase reporter plasmid used for ERα was ERE-luc and the plasmid HEG0 was used to express ERα (8). The Renilla luciferase

expression vector pRL-TK (Promega, Milan, Italy) was used as internal transfection control. For reporter gene assays, cells (1×10^5) were plated into 24-well dishes with 500 mL/well culture medium containing 5% FBS and transfected using X-treme GENE 9 DNA Transfection Reagent, as recommended by the manufacture (Merck Life Science, Milan, Italy), for 24 h with a control vector and HEG0, where required. A mixture containing 0.5 mg of reporter plasmid (ERE-luc) and 5 ng of pRL-TK was then transfected, after 8 h cells were treated with 100 nM E2 for additional 12 h. Luciferase activity was measured using the Dual Luciferase Kit (Promega, Milan, Italy) according to the manufacturer's recommendations. Firefly luciferase activity was normalized to the internal transfection control provided by the Renilla luciferase activity. Normalized relative light unit values obtained from cells treated with vehicle (−, ethanol, <0.1%) were set as 1-fold induction, upon which the activity induced by treatments was calculated. For gene silencing experiments, cells were plated onto 10-cm dishes and transfected by X-treme GENE 9 DNA Transfection Reagent for 24 h before treatments with a control vector and the plasmid DN/c-Fos encoding a c-Fos mutant that heterodimerizes with c-Fos dimerization partners but not allowing DNA binding (kindly obtained from Dr. C. Vinson, NIH, Bethesda, USA). The short hairpin RNAs used to silence ERα46 (targeting the C-terminal domain of the protein) were purchased from Merck Life Science (Milan, Italy) and the short hairpin RNAs used to silence IR were purchased from SABiosciences (Qiagen, Milan, Italy).

Chromatin Immunoprecipitation (ChIP) assay. Cells were grown in 10-cm dishes, exposed to treatments and then cross-linked with 1% formaldehyde and sonicated. Supernatants were immuno-cleared with salmon DNA/protein A-agarose (Merck Life Science) and immunoprecipitated with anti-c-fos antibody or nonspecific IgG. Pellets were washed, eluted with a buffer consisting of 1%SDS and 0.1 mol/L NaHCO3, and digested with proteinase K. DNA was obtained by phenol/chloroform extractions and precipitated with ethanol. The yield of target region DNA in each sample after ChIP was analyzed by real-time PCR. The primers used to amplify a region containing two AP-1 sites located into the IL11 promoter sequence were: 5′-CGTGAGCCCTGCAAGGCA-3′

(Fw) and 5′-GGGCGGGCGGGGGCAG-3′ (Rv). Data were normalized to the input for the immunoprecipitation and the results were reported as fold changes respect to nonspecific IgG.

Western blot analysis. Cells were grown in 10-cm dishes, exposed to treatments and then lysed in 500 μl RIPA buffer with protease inhibitors (1.7 mg/ml aprotinin, 1 mg/ml leupeptin, 200 mmol/liter phenylmethylsulfonyl fluoride, 200 mmol/liter sodium orthovanadate and 100 mmol/liter sodium fluoride). Samples were then centrifuged at 13,000 rpm for 10 min and protein concentrations were determined using BCA protein assay according to the manufacturer's instructions (Thermo Fisher Scientific, Milan, Italy). Equal amounts of whole-protein extract were resolved on 8-10% SDS-polyacrylamide gels and transferred to a nitrocellulose membrane (Merck Life Science, Milan, Italy), which were probed with primary antibodies against ERα (F-10; D-12), ICAM-1 (G-5), Insulin Rα (IR, N-20), phosphorylated ERK1/2 (E4), ERK2 (C-14), c-Fos (E-8) and β-actin (AC-15) (Santa Cruz Biotechnology, DBA, Milan, Italy), cyclin D1 (TA801655) (OriGene Technologies, DBA, Milan, Italy), p-IR (Y1146) (Cell Signaling Technology, Euroclone, Milan, Italy), IL11 (55169-1-AP) and integrin alpha 5 (10569-1-AP) (Proteintech, DBA, Milan, Italy). Proteins were detected by horseradish peroxidase–linked secondary antibodies (Bio-Rad, Milan, Italy) and then revealed using the chemiluminescent substrate for western blotting Clarity Western ECL Substrate (Bio-Rad, Milan, Italy). For subcellular fractionation studies, cells were lysed using 300 μl of cytosolic buffer (50 mM HEPES pH 7.5, 150 mM NaCl, 1% Triton X-100, 1.5 mM MgCl2, 1 mM EGTA, pH 7.5, 10% glycerol) with protease inhibitors (1.7 mg/ml aprotinin, 1 mg/ml leupeptin, 200 mmol/liter phenylmethylsulfonyl fluoride, 200 mmol/liter sodium orthovanadate and 100 mmol/liter sodium fluoride). Following centrifugation (14,000 g, 4 °C, 10 min), the supernatant was referred to as cytoplasmic fraction and the pellet containing nuclei was resuspended in high salt buffer (20 mM HEPES pH 7.9, 25% [v:v] glycerol, 420 mM NaCl, 1.5 mM MgCl2, 0.2 mM EDTA and protease inhibitors). For the extraction of nuclear proteins, the obtained solution was vortexed thoroughly, incubated overnight with agitation and centrifugated at 14000 g, 4 °C for 10 min. Equal amounts of

the collected supernatant, which represent the nuclear fraction, were then run on 10% SDS-PAGE and western blot analysis was performed as described above. The purity of the nuclear fraction was confirmed by immunoblotting with primary antibodies against β-actin (AC-15) and Lamin B1 (M-20) (Santa Cruz Biotechnology, DBA, Milan, Italy).

Co-immunoprecipitation assay. After exposure to treatments, cells were lysed and protein concentrations were determined using BCA protein assay, as described above. Proteins (200 μg) were then incubated for 2 h with 900 μl of immunoprecipitation buffer with inhibitors, 2 μg of anti-ERα (F-10), or anti-Insulin Rα (IR, N-20) antibodies and 20 μl of Protein A/G agarose immunoprecipitation reagent (Santa Cruz Biotechnology, DBA, Milan, Italy). Samples were then centrifuged at 13, 000 rpm for 5 min at 4 °C to pellet beads, which were washed four times with 500 μl of PBS and centrifuged at 13,000 rpm for 5 min at 4 °C. Supernatants were collected, resuspended in 20 μl RIPA buffer with protease inhibitors and 2X SDS sample buffer, and heated to 95 °C for 5 min. Samples were then run on SDS-PAGE gels, transferred to nitrocellulose and probed with primary antibodies. Western blot analysis and ECL detection were performed as described above.

Immunofluorescence microscopy. Cells were grown on a cover slip, exposed to treatments and then fixed in 4% paraformaldehyde in PBS, permeabilized with 0.2% Triton X-100, washed 3 times with PBS and incubated at 4 °C overnight with primary antibodies anti-cytokeratin-FITC (Beckman-Coulter, Milan, Italy), anti-FAPα (H-56) or ERα (F-10) (Santa Cruz Biotechnology, DBA, Milan, Italy). After incubation, the slides were extensively washed with PBS, probed with Alexa Fluor conjugated secondary antibodies (Thermo Fisher Scientific, Milan, Italy) for 1 h at room temperature. Finally, cells were washed three times with PBS, incubated with DAPI (4′,6-diamidino-2-phenylindole) (1:1000) for 3 min and, after washing, immunofluorescence images were obtained using the Cytation 3 Cell Imaging Multimode reader (BioTek, AHSI, Milan Italy) and analyzed by the Gen5 software (BioTek, AHSI, Milan Italy).

Electron immunocytochemistry. For immunogold staining, cell pellets were fixed in a mixture of

freshly prepared aldehydes (4% paraformaldehyde + 1% glutaraldehyde) in 0.1M phosphate buffer (pH 7.4) overnight at 4°C. After fixation, samples were washed several times in the same buffer over a period of 1 h, dehydrated through a series of graded ethanol baths, and gradually embedded in LR white acrylic resin. Ultrathin sections (60 nm) were prepared using a diamond knife and mounted on uncoated 300 mesh nickel grids. For indirect immunolabeling, grids with sections were floated on drops of 0.02M glycine in PBS at room temperature for 30 min to reduce nonspecific binding. Sections were then incubated with a primary antibody anti-ERα (1:50) in PBS 0.1% BSA at 4°C overnight. The grids were then washed in PBS, and transferred on small, 20 µL, drops of secondary antibody conjugated to 10-nm gold particles for 1 h. After immunolabeling, the sections were rinsed with PBS and distilled water, dried, and then examined with a "Jeol JEM-1400 Plus" electron microscope. Control staining to demonstrate immunohistochemical specificity included replacement of the primary antibody simply with PBS solution. Electron micrographs were taken at different magnification to study the entire cell profile and the subcellular distribution of each protein.

Enzyme-linked Immunosorbent assay. The concentrations of IL11 in supernatants from BCAHC-1 cells exposed to treatments were measured using human IL11 ELISA Kit (R&D Systems, Bio-Techne, Milano, Italy), according to the manufacturer's instructions. The plates were read at 450 nm on a Microplate Spectrophotometer Epoch™ (BioTek, AHSI, Milan Italy).

Proliferation assay. BCAHC-1 cells (1×10^4) were seeded in 24-well plates in regular growth medium, washed once they had attached, incubated in medium containing 2.5% charcoal-stripped FBS, transfected for 24 h (where appropriate) and then exposed to treatments. Transfection were renewed every 2 days and treatments every day. The proliferation rate was calculated counting the cells on day 5 using the Countess Automated Cell Counter, as recommended by the manufacturer's protocol (Thermo Fisher Scientific, Milan, Italy).

Colony formation assay. BCAHC-1 cells were cultured in regular growth medium to 90% confluence. Cells were then trypsinized, counted and seeded (1×10^3) in 6-well plates in medium

containing 2.5% charcoal-stripped FBS, transfected for 24 h (where appropriate) and then exposed to treatments, as indicated. Treatments were renewed every 3 days. After 10 days, cells were washed with PBS, fixed in acetone:methanol (1:1) for 3 min at room temperature and then stained with Giemsa for 10 min. A total of 10 pictures for each condition was detected using a digital camera and colony number was measured by ImageJ program.

Spheroid formation assay. For spheroid generation, 100 μL/well of BCAHC-1 cell suspensions (1 × 10^4) were dispensed into 2% agar coated 24-well plates in medium containing 2.5% charcoal-stripped FBS. Three days after seeding, tumor spheroids (a single spheroid per well) were exposed to treatments and a 50% medium and treatment replenishment was performed every 2 days. Images were obtained on day 20 using a conventional inverted microscope, thereafter cell number per spheroid was determined by trypsinizing three different spheroids, mixing the cell suspension with trypan blue and counting the number of viable cells. The total number of cells obtained was divided by the number of trypsinized spheroids.

***In vivo* studies.** The first group of female 45-day-old athymic nude mice (nu/nu Swiss, Envigo, Udine, Italy) (n = 6) was treated daily with vehicle (0.9% NaCl with 0.1% albumin and 0.1% Tween-20); the second group (n = 6) was treated daily with E2 (2.5 mg/kg/day); the third group (n = 6) was treated daily with insulin (Ins, 0.6 unit/day); the fourth group (n = 6) was treated daily with OSI-906 (50 mg/kg/day); the fifth group (n = 6) was treated once per week with ICI-182780 (ICI) (5 mg/kg/week); the sixth group (n = 6) was treated with OSI-906 (50 mg/kg/day) plus E2 (2.5 mg/kg/day); the seventh group (n = 6) was treated daily with OSI-906 (50 mg/kg/day) plus Ins (0.6 unit/day); the eighth group (n = 6) was treated with E2 (2.5 mg/kg/day) plus ICI-182780 (5 mg/kg/week); the ninth group (n = 6) was treated with Ins (0.6 unit/day) plus ICI-182780 (5 mg/kg/week). E2 was dissolved in ethanol, OSI-906 and ICI-182780 (ICI) were dissolved in DMSO and, finally, all treatments were resuspended in vehicle solution at work concentration. Mice well

tolerated all *in vivo* procedures, since no changes in body weight, motor function or food and water consumption were observed.

Histologic analysis and immunohistochemistry. Formalin-fixed, paraffin-embedded (FFPE) sections of tumor xenografts were cut at 5 μm, were mounted on slides precoated with poly-lysine, air dried, deparaffinized, rehydrated, (7–8 serial sections) and stained with hematoxylin and counterstained with eosin Y (Bio-Optica, Milan, Italy). Immunohistochemical experiments were performed after heat-mediated antigen retrieval. Hydrogen peroxide (3% in distilled water) was used, for 30 min, to inhibit endogenous peroxidase activity while normal goat serum (10%) was utilized, for 30 min, to block the non-specific binding sites. The epithelial nature of the tumors was verified by immunostaining with anti-human cytokeratin 18 antibody (1:100; Santa Cruz Biotechnology, Milan, Italy). Tumor sections were immune labelled with Ki67 (1:100) (DAKO, DBA, Milan, Italy), which served as a proliferation marker. All sections were incubated with primary antibodies at 4°C overnight. Then, a universal biotinylated IgG was applied (1:500) for 1 hour at room temperature, followed by ABC/HRP. Immunoreactivity was visualized by using DAB. For negative controls, nonimmune serum replaced at the same concentration of the primary antibody. Sections nuclei were also counterstained with hematoxylin. For each sample six-seven serial sections were processed and visualized using an OPTIKA XDS-3 microscope (OPTIKA Microscopes, Italy) and the images were acquired with OpticalSview software using an OPTIKA 4883.13 CAM.

Conditioned medium. BCAHC-1 cells were placed in medium without serum and treated with 100nM E2 and 10nM insulin for 4 h. Thereafter, BCAHC-1 cells were washed and fresh medium was added without serum for additional 8h. The supernatants were then collected, centrifuged at 3500 rpm for 5 min to remove cell debris and used as conditioned medium in the appropriate experiments.

Migration and invasion assays. Transwell 8 μm polycarbonate membranes (Costar, Sigma-Aldrich, Milan, Italy) were used to evaluate in vitro cell migration and invasion. 5×10^4 cells in 300 μL serum-free medium were seeded in the upper chamber coated with (invasion assay) or without (migration

assay) Corning® Matrigel® Growth Factor Reduced (GFR) Basement Membrane Matrix (Biogenerica, Catania, Italy) (diluted with serum-free medium at a ratio of 1:3). Medium containing 2.5% charcoal-stripped FBS was added to the bottom chambers in the presence of treatments. For the experiments with conditioned medium, it was added to the bottom chambers in the presence or absence of 200ng/ml Ab-IL11, where required. 8 h after seeding, cells on the upper surface of the membrane were then removed by wiping with Q-tip, and migrated or invaded cells were fixed with 100% methanol, stained with Giemsa (Sigma-Aldrich, Milan, Italy), photographed using Cytation 3 Cell Imaging Multimode Reader (BioTek, AHSI, Milan Italy) and counted using the WCIF ImageJ software.

TCGA database and data collection. Bioinformatics analyses were performed using the publicly available dataset The Cancer Genome Atlas (TCGA). The mRNA expression data (RNA Seq V2 RSEM) and the related patient clinical information of the Invasive Breast Cancer Cohort of the TCGA project were retrieved from UCSC Xena (https://xenabrowser.net/) on the 4th February 2021. Samples (n. 1247) were filtered for missing values and by the "sample type" in order to separate the tumor tissues (n. 1104) from the adjacent normal tissues (n. 113).

Survival analysis. The survival analyses were performed using the IL11 gene expression data of the TCGA patients along with the overall survival (OS), disease specific survival (DSS) and disease-free interval (DFI) information. The survivALL package was employed in R Studio (version 3.6.1) to examine Cox proportional hazards for all possible points of separation (low-high cut-points), selecting the cut-point with the lowest p-value [bioRxiv 208660], therefore dividing the patients with high and low IL11 expression levels. The Kaplan Meier survival curves were generated using the survival and the survminer packages.

Correlation and pathway analyses. The Pearson correlation coefficients (r-values) between the expression levels of IL11 and the other genes of the TCGA (n. 20530) dataset were assessed in R Studio using the *cor.test()* function. The statistical significance of the correlation coefficients was

calculated with the t-test, $p < 0.001$ was considered as a cut-off criterion. The first 1000 positively correlated genes were selected for the next evaluations. In order to investigate the biological significance of these genes, the Reactome package was employed in R to assess the signaling pathway enrichment analysis. The following parameters were used: organism = "human", p-value cut-off = 0.05, q-value cut-off = 0.2, p-AdjustMethod = "BH".

Statistical analysis. The statistical analysis was performed using ANOVA followed by Newman-Keuls' test to determine differences in means. Wilcoxon-Mann-Whitney test was used for the statistical comparisons for *in vivo* studies. Heatmaps were performed with the R pheatmap package. The t-tests, box plots and volcano plots were performed with the R tydiverse package. p-values < 0.05 were considered significant.

Supplementary References

1. Del Fabbro C, Scalabrin S, Morgante M, Giorgi FM. An extensive evaluation of read trimming effects on Illumina NGS data analysis. *PLoS One* 2013;8:e85024. doi: 10.1371/journal.pone.0085024.
2. Dobin A, Davis CA, Schlesinger F, Drenkow J, Zaleski C, Jha S, *et al.* STAR: ultrafast universal RNA-seq aligner. *Bioinformatics* 2013;29:15-21. doi: 10.1093/bioinformatics/bts635.
3. Pertea M, Pertea GM, Antonescu CM, Chang TC, Mendell JT, Salzberg SL. StringTie enables improved reconstruction of a transcriptome from RNA-seq reads. *Nat Biotechnol* 2015;33:290-5. doi: 10.1038/nbt.3122.
4. Anders S, Pyl PT, Huber W. HTSeq--a Python framework to work with high-throughput sequencing data. *Bioinformatics* 2015;31:166-9. doi: 10.1093/bioinformatics/btu638.
5. Love MI, Huber W, Anders S. Moderated estimation of fold change and dispersion for RNA-seq data with DESeq2. *Genome Biol* 2014;15:550. doi: 10.1186/s13059-014-0550-8.
6. Anders S, Huber W. Differential expression analysis for sequence count data. *Genome Biol* 2010;11:R106. doi: 10.1186/gb-2010-11-10-r106.

7. Lappano R, Rosano C, Santolla MF, Pupo M, De Francesco EM, De Marco P, *et al*. Two novel GPER agonists induce gene expression changes and growth effects in cancer cells. *Curr Cancer Drug Targets* 2012;12:531-42. doi: 10.2174/156800912800673284.
8. Bunone G, Briand PA, Miksicek RJ, Picard D. Activation of the unliganded estrogen receptor by EGF involves the MAP kinase pathway and direct phosphorylation. *EMBO J* 1996;15:2174-83.
