# Supplementary material for: Estrogen receptor variant ERα46 and insulin receptor drive in primary breast cancer cells growth effects and interleukin 11 induction prompting the motility of cancer‐associated fibroblasts
Source: Clin Transl Med. 2021 Nov 4;11(11):e516. doi: 10.1002/ctm2.516 (PMC8567034; doi:10.1002/ctm2.516)
Supplement: Supplementary file 5 — Supplementary TableS1 [file CTM2-11-e516-s002.pdf]

Supplementary Table 1. mRNA levels of hormone (A) and growth factor (B) receptors in BCAHC-1 cells, as evaluated by real-time PCR. AR, androgen receptor; ESR1, estrogen receptor α; ESR2, estrogen receptor β; GPER, g protein-estrogen receptor; GR, glucocorticoid receptor; MR, mineralocorticoid receptor; PGRA, progesterone receptor A; PGRB, progesterone receptor B; EGFR, epidermal growth factor receptor; ERBB2, Erb-B2 receptor tyrosine kinase 2; ERBB3, Erb-B2 receptor tyrosine kinase 3; IGF1R, insulin like growth factor 1 receptor; INSR A, insulin receptor A; INSR B, insulin receptor B. Values represent the mean ± SD of three independent experiments performed in triplicate. $p<0.05$.

A

**Gene**	**mRNA levels (pmol/µg total RNA)**
AR	< 0.001
ESR1 (C-E domains)	290 ± 23
ESR1 (A-B domains)	< 0.001
ESR2	< 0.001
GPER	< 0.001
GR	< 0.001
MR	< 0.001
PGRA	< 0.001
PGRB	< 0.001

B

**Gene**	**mRNA levels (pmol/µg total RNA)**
EGFR	< 0.001
ERBB2	< 0.001
ERBB3	< 0.001
IGF1R	< 0.001
INSR A	667 ± 35
INSR B	332 ± 29
