# Supplementary material for: Estrogen receptor variant ERα46 and insulin receptor drive in primary breast cancer cells growth effects and interleukin 11 induction prompting the motility of cancer‐associated fibroblasts
Source: Clin Transl Med. 2021 Nov 4;11(11):e516. doi: 10.1002/ctm2.516 (PMC8567034; doi:10.1002/ctm2.516)
Supplement: Supplementary file 6 — Supplementary TableS2 [file CTM2-11-e516-s005.pdf]

Supplementary Table 2. The top 100 up-regulated and down-regulated genes by E2 treatment along with their log2 fold change. $p \leq 0.05$.

Up-regulated genes	log2 fold change	Up-regulated genes	log2 fold change
YDJC	4.49	DUSP12	0.95
ITGB1BP2	4.26	CEP44	0.95
GDPD3	4.22	SS18L2	0.93
HIST1H2BE	3.65	CXCL2	0.93
FGF7	3.32	ST7L	0.93
IL11	3.3	TOR2A	0.93
OTOP1	3.13	TMEM171	0.92
PABPN1	1.98	SEPSECS	0.92
SAG	1.97	MIR3064	0.9
ZNF799	1.87	SSPN	0.89
MSRB3	1.65	RPPH1	0.89
FAM151B	1.62	F8	0.89
ZNF34	1.54	DIMT1	0.89
ELAC1	1.51	MB21D1	0.88
C11orf74	1.51	ALG5	0.88
P2RX3	1.51	PEX3	0.88
FGL1	1.5	HACL1	0.88
ZNF480	1.46	TXNRD2	0.88
FAM133CP	1.44	C8orf76	0.87
NEK4	1.41	TIMM10	0.87
ZKSCAN3	1.36	ZNF880	0.87
CFP	1.33	MTRNR2L6	0.86
PSTK	1.33	SNX16	0.86
MRPL57	1.26	MIR5047	0.86
TOMM5	1.25	VHL	0.84
ATF3	1.24	CRBN	0.84
TPPP3	1.21	C5orf22	0.84
ZNF564	1.15	MT2A	0.84
ZNF891	1.14	N6AMT2	0.83
SYT12	1.1	DHRS7	0.83
MTRNR2L2	1.1	DYNLL1	0.83
CCT6P3	1.1	UQCRC2	0.83
FBXL2	1.09	MRPL53	0.82
PIGH	1.08	PTDSS2	0.82
MIR100HG	1.07	MT1M	0.81
ZSCAN26	1.06	KBTBD8	0.81
SAC3D1	1.04	ARL1	0.81
MTRNR2L10	1.03	EIF4A2	0.81
CXCL1	1.03	ZNF625	0.81
TAF1B	1.02	CDK5RAP1	0.81
ZNF25	1.02	MED21	0.81
MT1B	1.01	GS1-124K5.11	0.8
MTRNR2L7	1.01	CLDN12	0.8
ZNF670	1.01	FASTKD2	0.8
MALAT1	0.99	MRPL15	0.8
MRTO4	0.99	ZNF878	0.8
NUDT19	0.98	NKAP	0.8
TMEM216	0.98	IFRD1	0.79
CDKN2AIPNL	0.97	PHOSPHO2	0.79
GNPNAT1	0.95	VKORC1	0.78

Down-regulated genes	log2_fold_change	Down-regulated genes	log2_fold_change
SNX29P2	-4.13	DAPK1	-0.67
RASD1	-3.54	TECR	-0.67
INPP5D	-3.18	ZNF628	-0.66
THBS2	-2.9	THAP1	-0.66
TNS1	-1.89	SHOX2	-0.66
IFIT3	-1.89	DNMBP	-0.66
LEF1	-1.31	RGS12	-0.65
MYB	-1.29	ZNF428	-0.65
ATP1A3	-1.16	MOV10	-0.65
ADD2	-1.12	PRSS22	-0.65
FOXC2	-1.09	ZFAT	-0.64
DCLK1	-1.08	PBX1	-0.64
CDC42EP2	-1.08	NR2F1	-0.64
SLCO4A1	-1.07	FBLN2	-0.63
FAM83D	-1.06	THBS1	-0.63
TNIK	-1.06	LAD1	-0.62
ITGA8	-1.05	TONSL	-0.61
AQP1	-1.04	IDH2	-0.61
ZNF341	-0.97	COL16A1	-0.61
ZFPM1	-0.92	LPAR2	-0.6
IRAK2	-0.91	CCNE1	-0.6
TYRO3P	-0.91	PML	-0.6
PANK1	-0.89	KSR1	-0.6
SEMA3B	-0.89	DBN1	-0.6
ARHGEF19	-0.88	PFKFB3	-0.6
HERC3	-0.84	TNS4	-0.6
PRND	-0.82	TSPAN9	-0.6
NRDE2	-0.81	FDPS	-0.6
CEP128	-0.81	PTOV1	-0.59
IGSF9	-0.8	MON1B	-0.58
DOCK6	-0.77	CDK20	-0.58
RNF135	-0.76	FDFT1	-0.58
ABCB8	-0.76	TMEM201	-0.58
PKMYT1	-0.75	NR1D1	-0.58
SLC4A8	-0.74	LOC494127	-0.58
C6orf132	-0.74	TNRC18	-0.57
MARCKSL1	-0.73	MFSD10	-0.57
CYP1B1	-0.73	FERMT1	-0.57
MICAL2	-0.73	IGFBP4	-0.57
AFAP1L2	-0.73	LSS	-0.56
CHTF18	-0.71	SMG9	-0.56
HOXA7	-0.71	PLEKHO1	-0.56
PEAR1	-0.69	PCYT2	-0.56
UHRF1	-0.68	KLF4	-0.56
PTP4A3	-0.68	MYBL2	-0.56
MEX3A	-0.68	HDAC6	-0.55
BCL9	-0.68	FIZ1	-0.55
SLC9A3R2	-0.68	WWP2	-0.55
EYA2	-0.68	SLC12A7	-0.55
VASH2	-0.68	RGL2	-0.55
